# The genome of the rice variety LTH provides insight into its universal susceptibility mechanism to worldwide rice blast fungal strains

**DOI:** 10.1016/j.csbj.2022.01.030

**Published:** 2022-02-10

**Authors:** Lei Yang, Mengfei Zhao, Gan Sha, Qiping Sun, Qiuwen Gong, Qun Yang, Kabin Xie, Meng Yuan, Jenny C. Mortimer, Weibo Xie, Tong Wei, Zhensheng Kang, Guotian Li

**Affiliations:** a State Key Laboratory of Agricultural Microbiology, Hubei Hongshan Laboratory, the Provincial Key Laboratory of Plant Pathology of Hubei Province, College of Plant Science & Technology, Huazhong Agricultural University, Wuhan 430070, China; b National Key Laboratory of Crop Genetic Improvement, Hubei Hongshan Laboratory, Huazhong Agricultural University, Wuhan 430070, China; c School of Agriculture, Food and Wine, University of Adelaide, Glen Osmond, SA, Australia; d Department of Plant Pathology and the Genome Center, University of California, Davis, CA 95616, USA; e State Key Laboratory of Crop Stress Biology for Arid Areas and College of Plant Protection, Northwest A&F University, Yangling 712100, Shaanxi, China

**Keywords:** *Oryza sativa*, LTH, Genome assembly, Nanopore, Plant immunity, Resistance gene

## Abstract

The widely used rice variety Lijiangxintuanheigu (LTH) shows a universal susceptibility to thousands of *Magnaporthe oryzae* isolates, the causal agent of devastating rice blast, making LTH an ideal line in resistance (*R*) gene cloning. However, the underlying genetic mechanism of the universal susceptibility has not been fully revealed because of the lack of a high-quality genome. Here, we took a genomic approach together with experimental assays to investigate LTH’s universal susceptibility to rice blast. Using Nanopore long reads, we assembled a chromosome-level genome. Millions of genomic variants were detected by comparing LTH with 10 other rice varieties, of which large-effect variants could affect plant immunity. Gene family analyses show that the number of *R* genes and leucine-rich repeat receptor-like protein kinase (LRR-RLK)-encoding genes decrease significantly in LTH. Rice blast resistance genes called *Pi* genes are either absent or disrupted by genomic variations. Additionally, residual *R* genes of LTH are likely under weak pathogen selection pressure, and other plant defense-related genes are weakly induced by rice blast. In contrast, the pattern-triggered immunity (PTI) of LTH is normal, as demonstrated by experimental assays. Therefore, we conclude that weak effector-trigger immunity (ETI)-mediated primarily by *Pi* genes but not PTI results in the universal susceptibility of LTH to rice blast. The attenuated ETI of LTH may be also associated with reduced numbers of *R* genes and LRR-RLKs, and minimally functional residual defense-related genes. Finally, we demonstrate the use of the LTH genome by rapid cloning of the *Pi* gene *Piak* from a resistant variety.

## 1 Introduction

Rice (*Oryza sativa*) is a major staple crop that feeds more than half of the world’s population [1]. However, devastating diseases caused by pathogens and pests pose a serious threat to rice production and thus global food security. Among them, the yield losses worldwide to rice blast caused by the fungal pathogen *Magnaporthe oryzae* is enough to feed more than 60 million people annually [2]. Cultivating disease-resistant varieties containing resistance (*R*) genes provides an economical and eco-friendly solution for sustainable agriculture. *R* genes play an important role in the plant immune system, consisting of pattern-triggered immunity (PTI) and effector-triggered immunity (ETI) [3], which are distinct but mutually dependent [4], [5]. PTI is triggered via recognition of microbial- or pathogen-associated molecular patterns (MAMPs or PAMPs), such as chitin and flagellin, by pattern recognition receptors (PRRs) on the surface of the cell [3]. This is turn leads to a rapid burst of apoplastic reactive oxygen species (ROS), providing initial defense against the pathogen. ETI is an intracellular response, triggered when *R* genes recognize corresponding pathogen-derived effectors. Most *R* genes encode nucleotide binding site-leucine rich repeat (NBS-LRR) proteins containing the nucleotide-binding adaptor shared by Apaf1, certain *R* genes and CED4 (NB-ARC) domain [6]. In rice, *R* genes against rice blast are called *Pi* genes [6], and *Pi* genes except for *Pi21, Pid2* and *Ptr* encode NBS-LRR proteins. Some *Pi* genes have to work in pairs, such as the *Pia* and *Pi-CO39* pair [7].

Lijiangxintuanheigu (LTH), a *japonica* rice variety from Yunnan Province of China, is universally susceptible to over 2,460 isolates of *M. oryzae* collected worldwide [8], and is widely used as a susceptible parental line in cloning *R* genes. Using LTH, Li et al. cloned an atypical *R* gene *bsr-d1*, which significantly improves rice blast resistance and maintains normal rice yield [9]. In a similar approach, more than ten *R* genes have been cloned, including *Pi57*(t), *Xa46*(t), *bph19*(t), *Pi60*(t), *Pi61*(t), *Pi-g*(t), *Pi36*(t), *Pi19*, *Pi-d*(t)*1* and *Pi-d*(t)*2* [9], [10], [11], [12], [13], [14], [15], [16]. In addition, a set of differential varieties has been developed in the LTH background as near-isogenic lines (NILs), each harboring a single *Pi* gene [17], [18]. The differential varieties have been widely used to distinguish pathotypes of worldwide *M. oryzae* isolates and assess the *M. oryzae* population genetic structure, which is valuable in monitoring the emergence of new pathotypes and therefore deploying resistant cultivars in disease management [19].

Accumulating genomic resources have greatly advanced functional studies in rice. For instance, the release of high-quality genomes of Nipponbare, 93–11 and KitaakeX has promoted *R* gene cloning [20], [21], [22], Stein et al have resolved the origin of rice and the events responsible for chromosomal infiltration [23], and Qin et al have revealed structural variants (SVs) associated with important agronomic traits in the rice pan-genome [24]. KitaakeX has a rapid life cycle (9 weeks) and has increasingly gained popularity in basic rice research [25]. Recently, the 3,010 rice genomes project provide short sequencing reads for LTH [26], which reveal substantial sequence polymorphisms in *R* genes, enabling detection of small polymorphisms but not SVs. However, characterizing *R* genes is still challenging without a high-quality genome, because *R* gene loci are highly diversified and inclined to SVs [27]. As a consequence, the efficient use of LTH and the accurate interpretations of LTH-related genomic and transcriptomic data are compromised by the unavailability of a reference genome. A high-quality genome of LTH will fulfill such a need for functional genomics studies and facilitate *R* gene cloning through either traditional mapping or whole-genome sequencing approaches.

In this study, we employed a genomics-driven approach together with experimental assays to investigate the nature of the universal susceptibility of LTH to rice blast. Using Nanopore long reads, we assembled the LTH reference genome with a scaffold N50 of 30.6 Mb and 51,800 gene models. In comparison to 10 commonly used rice varieties, we detected over 25 million of small variations and SVs. We found that the major characterized *Pi* genes are absent in LTH. The number of NB-ARC genes which are defined as *R* genes in our study and leucine-rich repeat receptor-like kinases (LRR-RLK)-encoding genes, a class of genes important for plant immunity, are significantly reduced in LTH. In addition, compared to *Brachypodium distachyon*, we found the median value of nonsynonymous substitution rate (Ka) to synonymous substitution rate (Ks) of *R* genes in LTH is the lowest among common rice varieties, and the ratio of Ka/Ks>0.25 in LTH is the smallest among the three *japonica* rice varieties. The selection pressure analysis demonstrates that the subpopulation to which LTH belongs received the lowest selection and harbored the highest degree of variation. Combined with the above results and the indigenous environment of LTH, *R* genes from LTH may be under weak selection pressure form rice blast. In addition, RNA-seq analysis shows that the number of induced plant defense-related genes is much lower in LTH than that in the resistant variety Ktiaake when challenged with *M. oryzae*. In the experimental assays, PTI of LTH is normal when challenged with chitin or flagellin22. The results together provide genomic insights into the mechanisms of the universal susceptibility of LTH to *M. oryzae*. The major reason for the universal susceptibility to rice blast of LTH is not PTI but attenuated ETI, which is caused by lack of *Pi* genes and may be also associated with reduced numbers of *R* genes and LRR-RLKs, and nonfunctional or minimally functional residual plant defense-related genes. With the LTH genome, we rapidly cloned the *Piak* gene, a new allele of the *Pi* gene *Pia*.

## 2 Materials and methods

### 2.1 Plant materials

Seeds of *Oryza sativa* subsp. *japonica* cv. Lijiangxintuanheigu (LTH) were surface-sterilized with HgCl_2_ (0.01%) and germinated on 1/2 Murashige and Skoog (MS) media in the growth chamber with a photoperiod of 12/12 h, temperature at 28 °C and light intensity of 280 μmol m^−2^ s^−1^. After one week, the seedlings were transplanted outside to the experimental station of Huazhong Agricultural University (Wuhan, China) in summer. The day/night period was around 14/10 h, and the temperature ranged from 30 to 35 °C. Leaves, stems, and panicles at different developmental stages from plants grown in the experimental station were collected in liquid nitrogen for RNA-sequencing. Roots for RNA-seq were collected from plants grown in Hoagland solution [28] in the growth chamber with the setting described above. Fresh leaves of 4-week-old plants were collected with liquid nitrogen for Nanopore sequencing.

### 2.2 Library preparation and genome sequencing

DNA isolation and genome sequencing were performed at Nextomics Biosciences (Wuhan, China). Briefly, high-quality genomic DNA was isolated from young leaves of 4-week-old seedlings using the cetyltrimethylammonium bromide (CTAB) method and purified with the QIAGEN genome kit (CAT# 13343) following the manufacturer’s protocol. DNA was quantified using a NanoDrop spectrophotometer (Thermo Fisher Scientific, USA) and accurately quantified using the Qubit 4.0 fluorometer (Invitrogen). Subsequently, LSK109 library construction kit (SQK-LSK109, Oxford Nanopore Technology) was used to construct DNA libraries following the manufacturer’s instructions, and DNA sequencing was performed on the high-throughput Nanopore sequencer PromethION (Chip model 9.4.1). The clean sequencing data were obtained by removing sequencing adaptors and low quality reads whose scores are less than seven from the original data. For next-generation sequencing, the genomic DNA was extracted from young leaves using the CTAB method and the sequencing was performed on the BGISEQ-500 platform (BGI, Shenzhen, China) following the manufacturer’s protocol. The raw data were filtered by removing sequencing adapters and low-quality reads (base quality less than or equal to five) for further analysis. The MDS plot of 3 k rice genomes was generated by the SNP seek web service [29].

### 2.3 Transcriptome sequencing and data analysis

Total RNA of multiple tissues from LTH, including leaves, shoots, roots, panicles and spikelets at different developmental stages, was extracted with Trizol [21]. The integrity of RNA was examined using agarose gel (1.5%) electrophoresis, and the concentration was measured on a NanoDrop spectrophotometer. The 2 × 150-bp paired-end sequencing was performed on the BGISEQ-500 sequencer (BGI, Shenzhen, China). The raw sequencing data were filtered by removing sequencing adapters, low-quality reads (base quality less than or equal to five) and reads with high percentage of unknown bases (‘N’ base > 5%), and the resultant clean data were stored in the FASTQ format for related analysis. For transcriptome assays of LTH and Kitaake infected by *M. oryzae* strain P131 [30], the leaf samples from two biological replicates were collected, and RNA was extracted and sequenced. The cleaned reads were aligned against the Nipponbare genome using HISAT2 [31]. Quantification of gene expression was performed using Featurecounts [32]. DESeq2 (version: 1.32.0) was used to assess differential expression between sample groups [33]. Differentially expressed genes (DEGs) were identified by applying a |Fold Change|>1 and false discovery rate (FDR) < 0.05. DEGs were functionally annotated using the GO database (http://geneontology.org/). Plant defense-related genes were retrieved from the funRiceGenes database [34].

### 2.4 Genome assembly, polishing, and scaffolding

The genome size of LTH was first estimated using *K*-mer frequency analysis and the 19-mer distribution was obtained using Jellyfish [35]. Before genome assembly, we first assessed the Nanopore sequencing data, the average read length for LTH is 15 kb with the longest of 292 kb, the read length N50 is 25 kb, and the average read quality score is 8.7 (Fig. S16 and Table S10). The final set of 1,633,102 Nanopore long reads were obtained by filtering with quality score (>7) and read length (>5 kb), and the 78x resulting high-quality reads were assembled in contigs using NextDenovo (version: 2.31, https://github.com/Nextomics/NextDenovo) (parameter: read cutoff = 15 k, seed cutoff = 21986) and NECAT with default parameters [36], and found that the NextDenovo assembly (N50 = 22.16 Mb with 27 contigs) is better than that of NECAT (N50 = 16.35 Mb with 132 contigs). The draft genome assembly from NextDenovo was polished for three rounds using Racon (version 1.4.19) [37] and one round using Medaka (version 1.2.1, https://github.com/nanoporetech/medaka) with the raw Nanopore long reads. Afterwards, we used NextPolish (version 1.3.1) [38] to polish the genome assembly for another three rounds with the paired-end short reads. After polishing, the contigs were assembled into scaffolds/chromosomes by aligning them to the Nipponbare genome using RAGOO (version 1.11) [39]. The genome assembly was further improved with the paired-end short reads using NextPolish. The assembly quality of the LTH genome was examined using the Benchmarking Universal Single-Copy Orthologs (BUSCO) (version 4.1.4) method [40]. The dot plot of LTH aligned to the KitaakeX genome was generated using D-GENIES [41].

### 2.5 Gene annotation

In order to fully annotate the protein-coding genes in the genome, we integrated ab initio prediction, homology searches and transcript-based prediction. We performed the ab initio prediction and the homology-based prediction of gene models using Braker2 [42] with the support of RNA-seq data from multiple tissues as well as the UniProt protein database [43]. For transcript-based prediction, we used HISAT2 [31] and StringTie [44] to align and assemble the transcripts. We also used Trinity [45] to *de novo* assemble transcripts and conducted genome-guided assembly of transcripts using the BAM file resulted from HISAT2. The Program to Assemble Spliced Alignments (PASA) pipeline (version: 2.4.1) was used to integrate all of the transcripts predicted above [46]. Afterwards, the coding region within the transcripts was identified using TransDecoder (version 5.5.0, https://github.com/TransDecoder/TransDecoder). Based on the above results, we used EVidenceModeler (EVM) to generate the consensus gene models and used the PASA pipeline for further pruning the annotation [47].

### 2.6 Identification of TE-Related genes

Transposable element (TE)-related genes were identified by aligning the annotated genes to a TE library using BLASTN and screening the genes with an E value < 1e-10. The TE library is composed of the specific LTH TE library produced by EDTA [48] and Gramineae TE library.

### 2.7 Gene function annotation

The predicted gene modules were first aligned to the EGG-NOG database [49] to achieve the best Orthologous Groups and then the best-matched Orthologous Groups were functionally annotated using updated versions of Gene Ontology [50], KEGG pathways [51], SMART/PFAM domains [52] and KEGG modules. Gene functions were further annotated according to the best-matched Orthologous Groups.

### 2.8 Noncoding RNA prediction

The five different types of noncoding RNA genes including tRNA, rRNA, snoRNA, snRNA, and miRNA were predicted using *de novo* and homology search methods. We used tRNAscan-SE [53] to identify tRNAs with default parameters. The snoRNAs, snRNAs and miRNAs were annotated using INFERNAL from the Rfam database [54]. The rRNAs were searched using RNAmmer [55].

### 2.9 Repeat annotation

RepeatMasker-open-4.0.7 (http://www.repeatmasker.org) was used to identify the repetitive sequences in the LTH genome. The repeat databases used were Dfam_Consensus-20170127 [56] and RepBase-20170127 (http://www.girinst.org/).

### 2.10 Analysis of genome collinearity

The JCVI tool was used to analyze the collinearity of LTH, KitaakeX and Nipponbare with default parameters (--cscore = 0.7, -–no_strip_names) [57].

### 2.11 Analysis of genomic variations

SNPs and InDels: we used Burrows-Wheeler Aligner (BWA) [58] to align the paired-end short reads of LTH to the KitaakeX genome using default parameters and then used Picard (http://broadinstitute.github.io/picard/) in GATK [59] (version 4.18) to mark the duplicates. Afterwards, SNPs and InDels were called using bcftools (version 1.11–21-g0987715) with parameters (- mv filter - i 'DP > 20 && QUAL > 40′) [60]. The effect of variations on genes was annotated using SnpEff with default parameters [61]. The same protocol was used to call and annotate SNPs and InDels between LTH and Nipponbare [62], KitaakeX [21], 93–11 [24], Shuhui498 (R498) [63], IR8, IR64 [24], N22 [24], ZH11 [64], *O*. *rufipogon* as well as *O. nivara* [23].

Structural variants: we used NGMLR (version 0.2.7) to align the Nanopore sequencing data to the commonly used rice varieties and then used Sniffles (version 1.0.12b) to call the structural variants with default parameters [65]. To simplify the results, complex events such as deletions coupled with inversions were classified as deletions and inversions coupled with duplications as inversions. Structural variants were visualized using the Integrative Genomics Viewer (IGV) [66]. The breakpoints of the largest inversion on chromosome eight were identified using the Sniffles tool and verified using PCR.

### 2.12 Genomic feature visualization

To visualize our genome annotation results, we used the following methods: (i) using bedtools (version 2.29.2) [67] to generate 100-kb windows of the genome; (ii) the GC content and repeat content were calculated using seqtk (version 1.3-r106) (https://github.com/lh3/seqtk); (iii) the density of SNPs, InDels and collinearity blocks were generated using our internal scripts and the gene expression was calculated from the RNA-seq data using bedtools. Finally, all of the above results were visualized using Circos (version 0.69–8) [68].

### 2.13 Gene family analysis

We used the following methods to identify different gene families from rice genomes. The proteins of rice varieties KitaakeX, Nipponbare, 93–11, R498, and IR8 [21], [23], [62], [63] were aligned against the raw Hidden Markov Model (HMM) of the NB-ARC domain (PF00931), the WRKY DNA -binding domain (PF03106), and the protein kinase domain (PF00069) using HMMER [69] with default parameters. To identify proteins in the LRR-RLK family, protein kinases were further classified by leucine-rich repeat (LRR) domains (PF00560, PF07723, PF07725, PF12799, PF13306, PF13516, PF13855, PF14580 and PF18805) [70]. (iii) The glycoside hydrolases, glycosyltransferases and carbohydrate-binding modules were identified using the dbCAN pipeline [71]. The E-value of all the above analysis is 1e-5. We further analyzed the *R* genes in detail. The distribution of *R* genes on each chromosome of LTH and KitaakeX was modified from a map generated using MapGene2Chrom [72].

To verify the genomic results regarding *R* gene losses in LTH, we fetched the genomic sequences of these genes from KitaakeX using bedtools and seqtk. The primers were designed using Primer-Premier 5 [73], and the PCR products were separated and visualized though agarose gel electrophoresis.

### 2.14 *R* gene family and phylogenomic analysis

*R* gene families were clustered using OrthoFinder (version 2.2.7) [74] with parameters (-S diamond -M msa). A species tree was inferred from the orthogroups with a single-copy ortholog for each genome using RaxML (version 8.2.12) [75]. Based on a calibration of divergence times using the *O. rufipogn* and *B. distachyon* (>42 and < 52 Mya) divergences, the divergence time for the inferred species tree was calculated using MCMCtree implemented in PAML (version 4.9j) [76]. Gene families inferred from OrthoFinder were used to calculate the expansion or contraction of the gene families in each lineage using CAFÉ (version 4.2.1) [77]. The Ka/Ks value was calculated using Tbtools [78] with the paired orthogroups form OrthoMCL (version 2.0.9) [79]. The phylogenetic tree was generated using FastTree (version 2.1.10) [80] and visualized using FigTree (Version:1.4.4, http://tree.bio.ed.ac.uk/software/figtree/).

### 2.15 Selections pressure analysis

The 29 million biallelic SNPs were downloaded from SNP-Seek (https://snp-seek.irri.org/_snp.zul), and SNPs with a minor allele frequency of < 0.05 were removed using plink (Version 1.9, https://www.cog-genomics.org/plink2/). The nucleotide diversity (π) and fixation index (Fst) were performed on the filtered SNP set using VCFtools (Version 0.1.17) [81], and the linkage disequilibrium analysis was calculated using PopLDdecay (Version 3.41, https://github.com/BGI-shenzhen/PopLDdecay).

### 2.16 Analysis of *Pi* and atypical *R* genes

We selected 43 published *Pi* proteins (Table S9) including Pb1, OsRGA5, Pi1-5, Pi1-6, Pi2, Pi5-1, Pi5-2, Pi7-1, Pi7-2, Pi9, Pi21, Pi36, Pi37, Pi64, Pi35, Pi57, Pi50_NBS4_3, Pi50_NBS4_1, Pi54, Pi56, Pi63, Pia, Pib, Pid3, Pid4, Pigm-R6, Pii-1, Pii-2, Pik-1, Pik-2, Pik-h, Pikm1, Pikm2, Pikp-1, Pikp-2, Piks-1, Piks-2, Pish, Pit, Pita, Pizh, Piz-t, and Ptr and 3 atypical proteins/genes which are involved in rice blast resistance including Bsr-k1, *Bsr-d1* and Pi-d2. The amino acid sequences of some Pi proteins without accession numbers were extracted from publications [82], [83]. We used BLASTP to align these proteins to the protein set of the LTH genome. We filtered those hits whose identity is<75% and E-value > 1e-10.

### 2.17 Measurement of reactive oxygen species

We measured the ROS following chitin treatment following a previous protocol [84] using the selected rice varieties including Nipponbare, Kitaake, LTH, IR8, 93-11, and R498. Briefly, surface-sterilized rice seeds were germinated and grown on 1/2 MS media in the growth chamber for 14 days. The top of the second leaf of the seedling was cut into small leaf disks (3 mm × 3 mm). The leaf disks were put into a 96-well plate with water under light overnight. The ROS measurement procedure was done with 6 mM chitin (GLPBIO) and 10 μM flagellin22. The chemiluminescence indicating ROS was measured on a multimode plate reader (SPARK-10 M, TECAN). Four biological replicates, each of which has two technical replicates, were used for each sample. Distilled water was used as the mock control.

### 2.18 Gene cloning of *Piak*

We constructed the F2 segregating population by crossing Kitaake with LTH. The F1 plants were verified using primers *OsMADS50*/5FR (Table S11). In the plant infection assay, *M. oryzae* strain P131 was cultured on the oatmeal agar plate (OTA) at 28 °C for 10 days and fresh mycelial plugs (2 mm × 2 mm) were used to inoculate the 5-week-old F2 plants using the punch method [9]. The punched leaf area was sealed with clear Scotch tapes for 10 days before the disease symptom was examined. Primers for genotyping of different *Pi* loci, including *Pia*, *Pish*, *Pi-cd* and *Pi37* are shown (Table S11).

The *AVR-Pia* gene [85] including its native promoter was amplified from *M. oryzae* strain P131 with primers AVR-F/R and cloned into vector PKNTG [86]. The resultant construct was sequenced and transformed into *M. oryzae* strain ZB25 which does not harbor the *AVR-Pia* gene. *M. oryzae* protoplast preparation and transformation were performed as described [87]. Positive transformants confirmed using PCR were used to infect rice Kitaake and LTH as described above.

## 3 Results

### 3.1 Assembling a high-quality genome of LTH

**Fig. 1:**
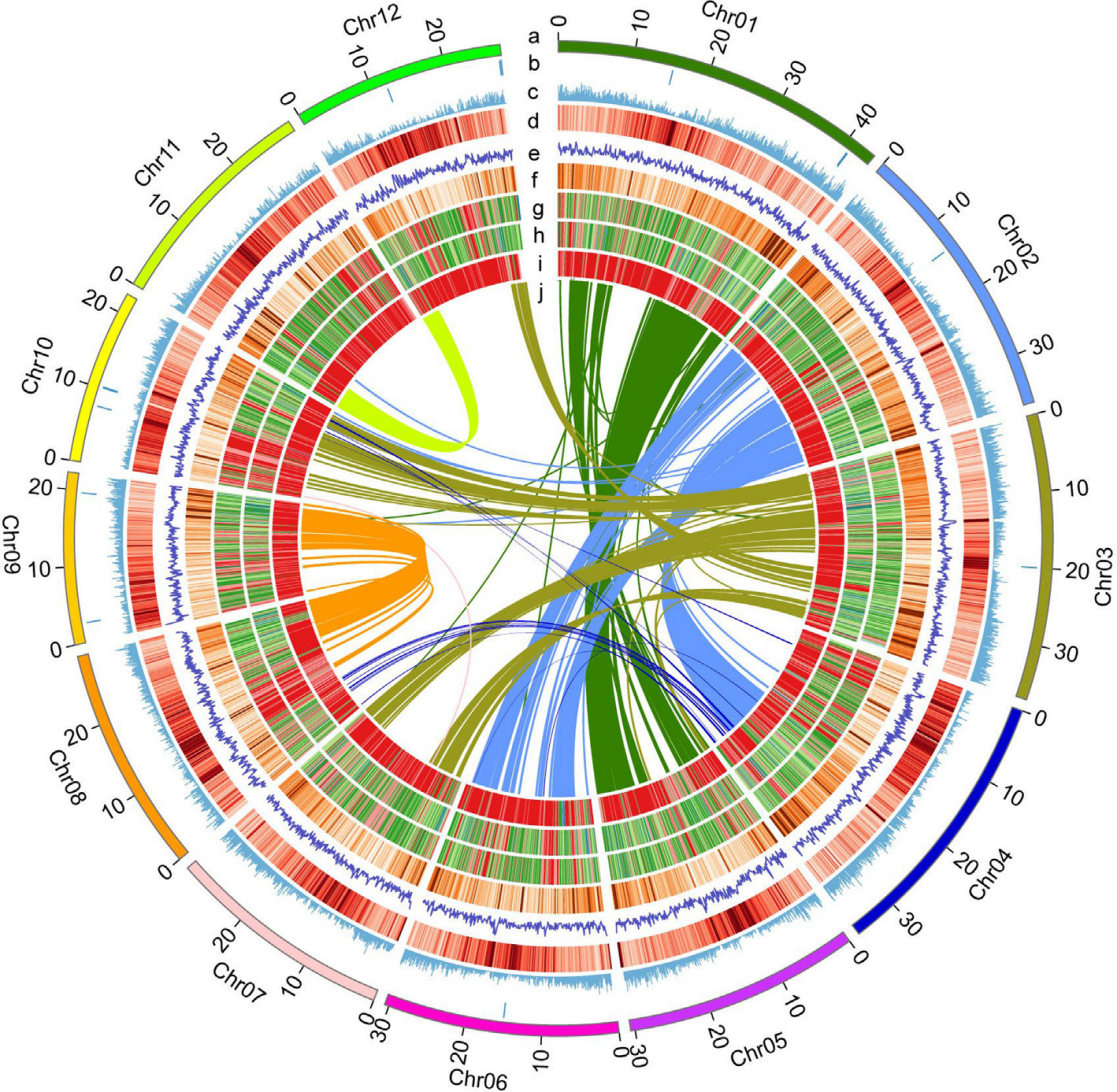
The Lijiangxintuanheigu (LTH) genome and its comparisons with different rice varieties. (a) The 12 LTH chromosomes on a Mb scale. (b) Contig gaps. (c) Gene density. (d) Repeat density. (e) GC content. (f) Gene expression level. SNPs and InDels of LTH compared to *japonica* KitaakeX (g), Nipponbare (h), *indica* 93–11 (i). (j) Homologous genes in the LTH genome. Results are displayed in nonoverlapping 100-kb intervals.

The widely used rice variety LTH belongs to the *japonica* group based on phylogenetic analysis of genome-wide single-nucleotide polymorphisms (SNPs) from the 3,010 rice accessions (Fig. S1) [29]. Prior to the genome assembly, using the 22.7-Gb next-generation sequencing (NGS) data, we estimated the genome size as 383 Mb according to the *k*-mer analysis (Fig. S2), which is comparable to other *japonica* rice varieties [62]. We took a hybrid strategy in assembling the LTH genome with Nanopore long reads and NGS short reads. A total of 29.2-Gb Nanopore long reads (around 80 × in coverage) were assembled into contigs. We further polished the draft genome with the Nanopore long reads and NGS short reads. The final assembly consist of 27 contigs with a contig N50 of 22.7 Mb and the largest contig of 35.8 Mb (Table 1). Using the RAGOO scaffolding approach [39], these contigs were anchored into 12 chromosome-scale (N50, 30.6 Mb) pseudomolecules and 5 contigs were already at the chromosome level, including chromosomes 4, 5, 7, 8 and 11 (Fig. 1a, 1b and Table S1). At completion, the assembled genome is 374.5 Mb, which covers 98% of the estimated genome.

**Table 1:** Genomic features of LTH.

Genomic feature	
Genome coverage	78X
Contigs	27
Total bases in contigs (Mb)	374.5
Largest contig (Mb)	35.8
Contig N50 (Mb)	22.7
L50	7
Gene models	51,800
Non-TE genes	36,816
GC content (%)	43.42
Repeat content (%)	46.08
BUSCO completeness (%)	99.00
Mapping rate for short reads (%)	99.87

We evaluated the completeness of the assembly at the gene space using Benchmarking Universal Single-Copy Ortholog (BUSCO) analysis. The result showed that the assembly covered 99.0% of the 1,614 core genes in the embryophyta_odb10database (Table S2), which is comparable to two “gold standard” rice reference genomes, the *japonica* variety Nipponbare (98.9%) and the *indica* variety Shuhui498 (R498) (98.6%) (Table S2) [63]. We aligned the NGS reads to the LTH genome, resulting in a mapping rate of 99.87% (Figure S3), which further indicates the high-quality of the reference genome. To obtain an accurate gene set in the LTH, 6.26-Gb RNA-sequencing (RNA-seq) data were generated from five tissue types, including leaves, shoots, roots, panicles and seeds with each at different developmental stages. We used an integrated strategy for gene prediction, including ab initio prediction, homology searches and transcript-based prediction to annotate protein-coding genes in LTH. We predicted 51,800 gene models and 89.68% of them were supported by the RNA-seq data. Of these gene models, 36,816 were classified as non-transposable element (non-TE) gene loci. On the protein level, the BUSCO analysis using the 3,236 single-copy genes indicates that 96.1% of them were found in the annotated genes completely (Table S3), slightly higher than the BSUCO scores of two recently published high-quality rice genomes [88]. We functionally annotated the gene models, and 30,737 genes (59.3%) showed hits in the InterPro database [89]. We further annotated noncoding RNAs using homology searches, and identified 784 transfer RNA (tRNA) genes, 226 ribosomal RNA (rRNA) genes, 85 small nuclear RNA (snRNA) genes, 612 small nucleolar RNA (snoRNA) genes and 1,919 microRNA genes (miRNA) (Table S4 and Supporting data 1). Gene density analysis showed that the majority of gene models are located towards the chromosomal ends (Fig. 1c), similar to other rice genomes [90].

Based on homology searches, a total of 172.81 Mb of the genome was masked as repetitive sequences (Fig. 1d). The proportion of repetitive sequences (46.14%) in LTH genome is higher than that in Nipponbare (39%). Among these repetitive sequences, the long-terminal repeat (LTR) element family is the highest (23.18%), followed by DNA transposons (16.88 %). Further classification of LTRs shows that Gypsy has the largest number (18.98%), followed by Copia (3.69%) (Table S5). We used the LTR Assembly Index (LAI) to evaluate the assembly quality at the intergenic and repetitive space, and the LAI score of LTH is 21.20, comparable to the Nipponbare genome (22.01). Taken together, these results including the contig N50 of 22.7 Mb, the genomic BUSCO score of 99% and the LAI score above 20 indicate that the assembly of the LTH genome reaches the “gold standard” level [91].

### 3.2 Whole-genome comparisons of LTH to other rice varieties

**Fig. 2:**
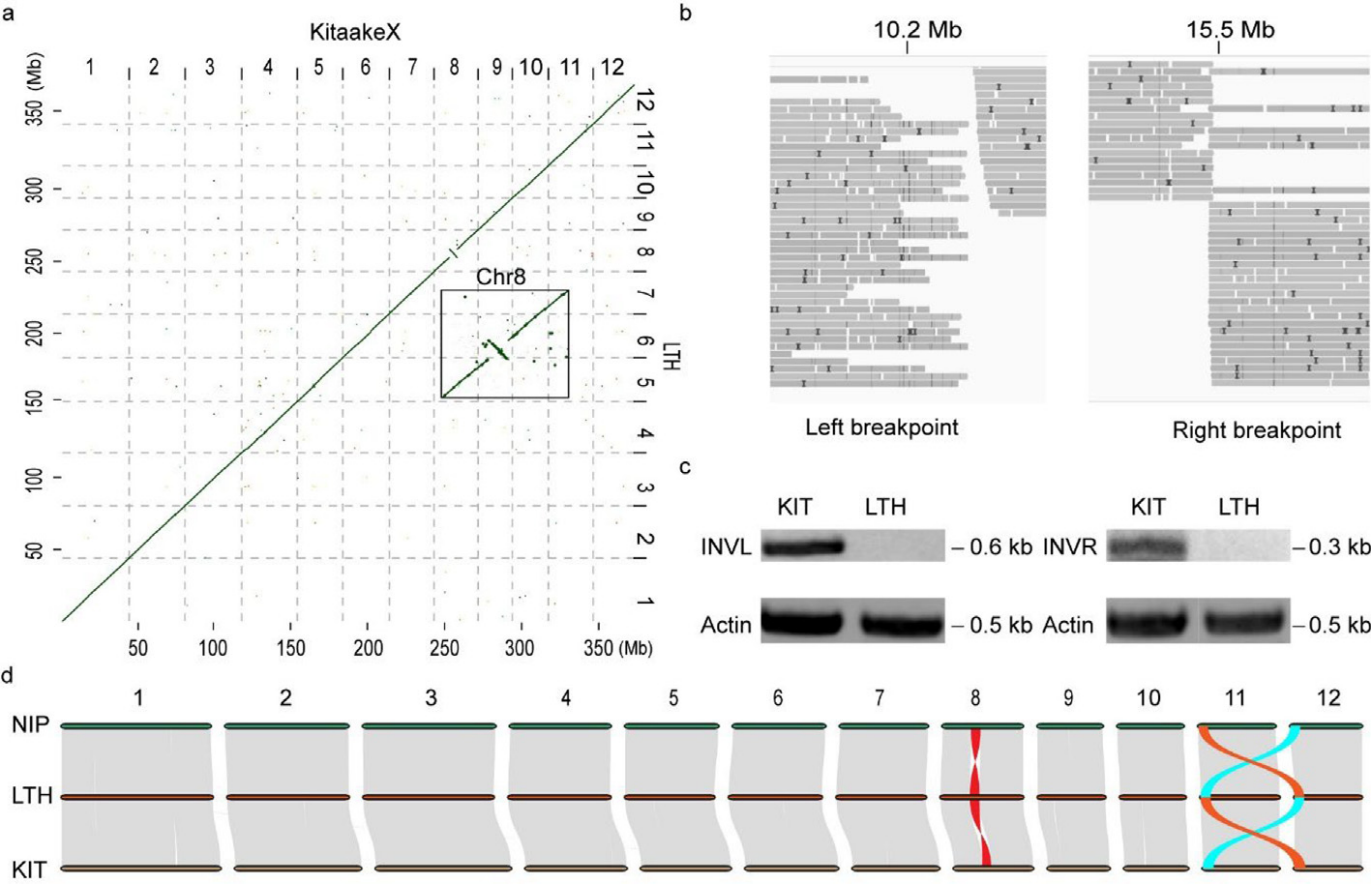
Genome synteny of Lijiangxintuanheigu (LTH) to other model rice varieties. (a) Dot plot comparing the LTH and KitaakeX (KIT) genomes. Shown are alignment blocks with>75% sequence similarity. The inset highlights the 5.3-Mb inversion on chromosome 8. (b) IGV screenshots of the breakpoints of the 5.3-Mb inversion using the Nanopore long reads. (c) PCR verification of the 5.3-Mb inversion. Primer pairs were designed on the two sides of each breakpoint. The actin amplicon was used as the control. (d) Chromosome-level collinearity patterns between Nipponbare (NIP), LTH, and KitaakeX, highlighting the inversion (red) on chromosome 8 and translocation events (brown and turquoise) on chromosomes 11 and 12. Each line represents a syntenic block with similarity of 70% or more. (For interpretation of the references to color in this figure legend, the reader is referred to the web version of this article.)

We aligned the LTH assembly to the genomes of model rice varieties Nipponbare and KitaakeX using the genome sequences [21]. Dot plots show that there are high linear relationships between the genomes, with the exception of some large variations on chromosomes 8, 11 and 12 (Fig. 2a and S4). There is a 5.3-Mb inversion on chromosome 8, which is also supported by the Nanopore long reads visualized using Integrative Genomics Viewer (IGV) (Fig. 2b). We further confirmed this inversion using PCR (Fig. 2c). Using the recently published rice pan-genome, we aligned the LTH genome to other rice genomes and observed only another temperate *japonica* rice variety 002,428 having this inversion [24] (Supporting data 2). We conducted a collinearity analysis on the coding sequence (CDS) of Nipponbare, LTH, and KitaakeX (Fig. 2d). The collinearity analysis showed that the three genomes have a highly linear relationship except for the inversion on chromosome 8. We also found a special linear relationship between chromosomes 11 and 12 (Fig. 2d), which may be related to the previously published tandem duplication event in the rice genome [92].

With the completion of the high-quality LTH genome, we analyzed the genomic differences between LTH and other rice varieties. We compared LTH to Nipponbare and KitaakeX genomes to identify genomic variations including SNPs and insertions/deletions (InDels) using samtools and bcftools based on read mapping (see Methods). We identified 706,477 SNPs and 85,440 InDels between Nipponbare, and 712,949 SNPs and 91,937 InDels between KitaakeX and LTH. We also compared LTH to 8 commonly used rice varieties and called total 20,547,906 SNPs and 2,541,290 InDels (Supporting data 3). We used Sniffles to analyze SVs between LTH and other rice genomes (Supporting data 4). Comparing with KitaakeX, we called a total of 15,065 SVs including 1,359 translocations, 7,905 deletions, 454 duplications, 4,982 insertions and 365 inversions. The SV results also called the inversion on chromosome 8 (Table S6), further proof of the above results.

We functionally annotated the genomic variations, focusing on Nipponbare and KitaakeX, and found that there are 83,971 missense variations and 3,137 nonsense variations for Nipponbare caused by SNPs, and 34,867 missense variations and 555 nonsense variations for KitaakeX (Figure S5). Further classification of these variations predicted that 7,304 and 2,277 variations in Nipponbare and KitaakeX (Figure S6), respectively, have significant impacts on gene functions. We focused on the *R* genes. Compared to Nipponbare and KitaakeX, LTH has 72 and 76 *R* genes affected, respectively (Table S7 and Table S8).

### 3.3 Gene family analysis of LTH and other rice varieties

**Fig. 3:**
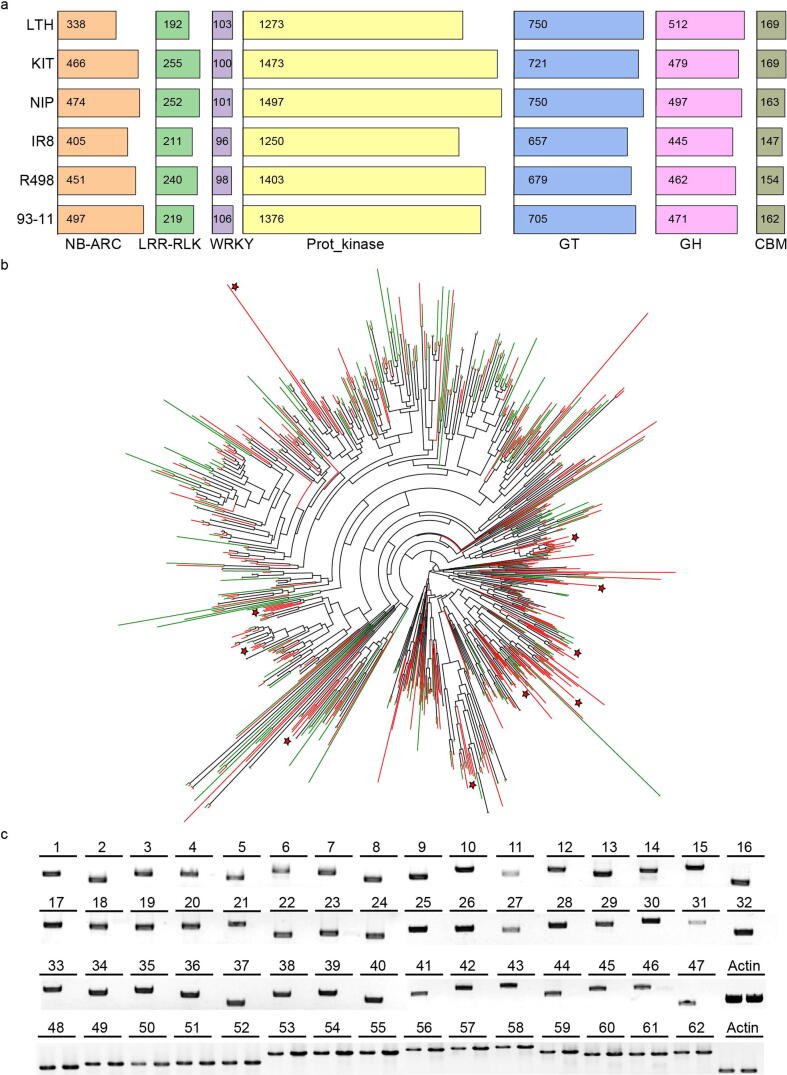
Gene family analysis of Lijiangxintuanheigu (LTH) and other rice varieties. (a) Comparison of different gene families in six rice varieties. The gene families include NB-ARC (Nucleotide-Binding adaptor shared by Apaf1, certain *R* genes and CED4), LRR-RLK (leucine-rich repeat receptor-like protein kinases) and WRKY transcription factors, GT (glycosyltransferases), GH (glycoside hydrolases) and CBM (carbohydrate-binding modules). (b) Maximum-likelihood tree of the NB-ARC domain-containing proteins in KitaakeX (red) and LTH (green). The red star indicates the clade that lacks LTH genes. (c) PCR validation of *R* genes absent from LTH. PCR amplicons were visualized on 1% agarose gels. For each *R* gene, the left lane indicates Kitaake (Kit), and the right lane LTH. (For interpretation of the references to color in this figure legend, the reader is referred to the web version of this article.)

To fully illuminate the genetic nature of LTH’s susceptibility to rice blast, we compared multiple gene families of LTH to high-quality genomes of Nipponbare, KitaakeX, 93–11, IR8 and R498 using the same pipeline [21], [23], [62], [63], which have high-quality genomes and are commonly used as research varieties. These families included NB-ARC, LRR-RLK and WRKY, which are often involved in plant immunity, glycoside hydrolases (GH), glycosyltransferases (GT) and proteins containing carbohydrate-binding modules (CBM), which are more related to plant growth and development. We found that compared with these rice varieties, LTH contains only 338 *R* genes while other rice varieties contain>400 *R* genes (Fig. 3a). We also found the number of LRR-RLKs in LTH is smaller than other rice varieties, and LTH has 60 fewer LRR-RLKs than Nipponbare and KitaakeX, whereas the number of WRKY transcriptions factors is comparable. As a control, genes usually involved in plant growth and development, not immunity, had similar numbers in LTH as compared to other rice varieties.

We next compared the evolutionary trajectories of predicted *R* genes in LTH and KitaakeX using phylogenetic analysis. Most *R* genes are categorized into different clades. Compared to KitaakeX, LTH lacks multiple clades (Fig. 3b). We mapped the *R* genes along the chromosomes of LTH and KitaakeX and observed that *R* genes of LTH are fewer than that of KitaakeX on each chromosome with the largest difference on chromosomes 11 (Figure S7), which also harbor the most *R* genes among the 12 chromosomes. To verify this, we selected 76 *R* genes predicted to be absent in LTH compared to Kitaake for PCR detection and successfully verified 47 of them (Fig. 3c). We also performed PCR analyses of 15 *R* genes common to LTH and Kitaake as controls (Fig. 3c).

**Fig. 4:**
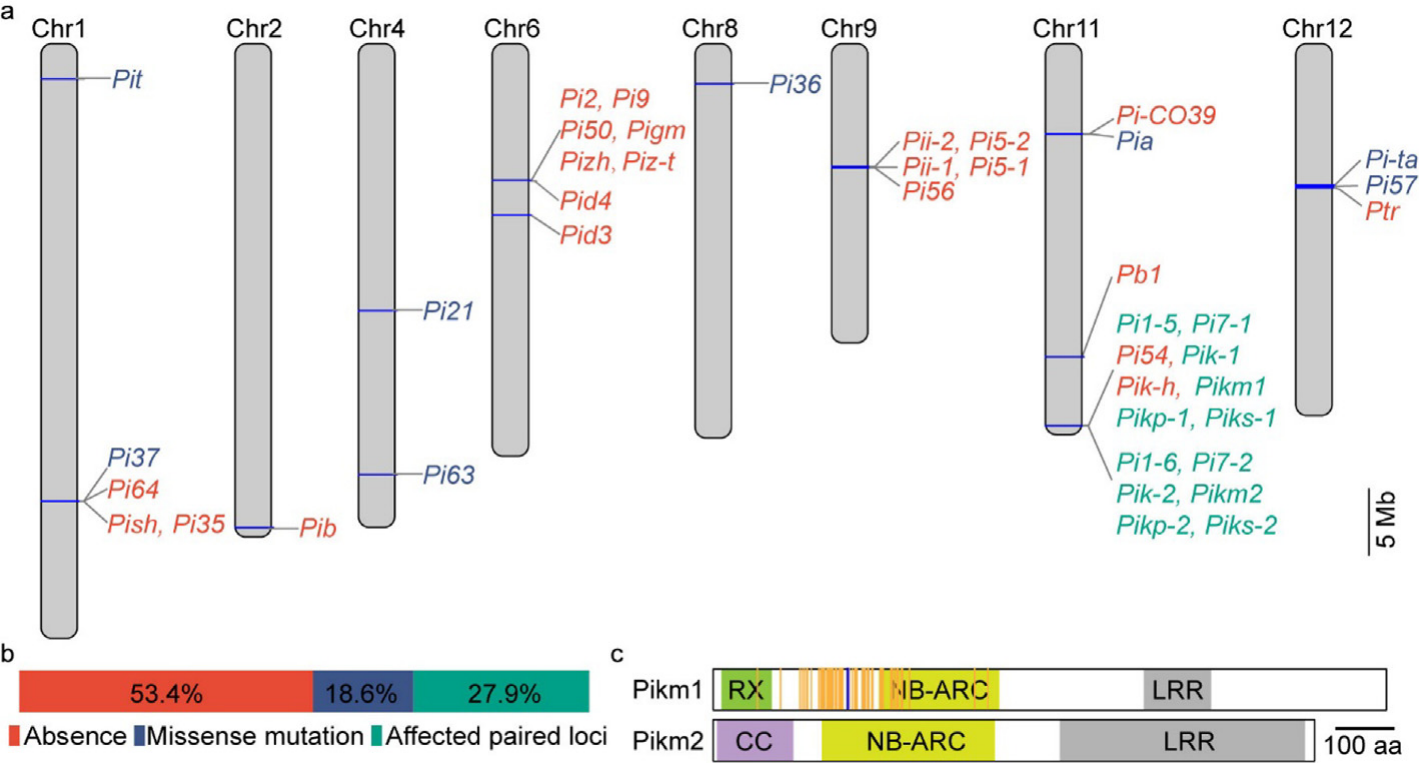
Analysis of *Pi* genes in the Lijiangxintuanheigu (LTH) genome. (a) *Pi* genes and related alleles affected by different types of variations in the LTH genome. Red, absence; dark blue, missense mutation; green, one gene in the working pair is functional and the other nonfunctional. (b) Different types of variations that affect *Pi* genes. (c) An example of paired *Pi* genes in the LTH genome. RX, potato virus X resistance protein; CC, coiled-coil domain; NB-ARC, nucleotide-binding adaptor shared by Apaf1, certain *R* genes and CED4; LRR, leucine-rich repeat. Amino acid polymorphisms: orange, amino acid substitution; blue, insertion. (For interpretation of the references to color in this figure legend, the reader is referred to the web version of this article.)

*Pi* genes, which are mostly of the NBS-LRR gene class, are important for blast resistance in rice. We used 43 available *Pi* genes including different alleles to search the LTH genome (Table S9). The sequence alignment of these *Pi* genes is shown (Figure S8), and the majority of *Pi* genes have no or very low homology in LTH. To further analyze these genes according to the genomic variations, we classified *Pi* genes into three categories (Fig. 4a and 4b). The first category is “absence”, in which the homologous region of the LTH gene is<75% [93] of the published *Pi* gene or the aligned length is much shorter than the *Pi* gene. There are 23 *Pi* genes in this category, including *Ptr, Pi5-1, Pi5-2, Pizh-1, Piz-t, Pi50_NBS4_1, Pish, Pi50_NBS4_3, Pi35, Pi64, Pi2, Pigm-R6, Pi9, Pib, Pi-CO39, Pid3, Pi56, Pb1, Pi54, Pid4, Pik-h, Pii-2 and Pii-1*. The second category includes truncated Pi proteins and Pi proteins with altered amino acids, including Pita, Pi57, Pi36, Pi63, Pi37, Pi21, Pia and Pit. Among these, the Pita protein in the LTH genome (889 amino acids) is shorter than the functional allele (928 amino acids), lacking the key 918th amino acid residue [94]. The Pia and Pi37 proteins in LTH genome harbor 41 and 169 altered amino acid residues, respectively, resulting into nonfunctional proteins. Pi21 and Pit proteins in LTH are the same as the susceptible alleles [95], [96]. The third category includes predicted functional alleles whose functions are nullified by their nonfunctional working pairs. This category includes 6 *Pi* genes of 3 working pairs. *Pikm2, Pik-2, Piks-2* in LTH are identical to the published functional alleles [97], [98]. However, the nonfunctional Pikm1 allele of LTH dismantles the resistance conferred by this pair of genes (Fig. 4c). The same situation occurs for *Pik1/2* [98] as well as *Piks-1/2* and possibly for the *Pi1-5/6*, *Pikp-1/2*, and *Pi7-1/2 R* gene pairs.

**Fig. 5:**
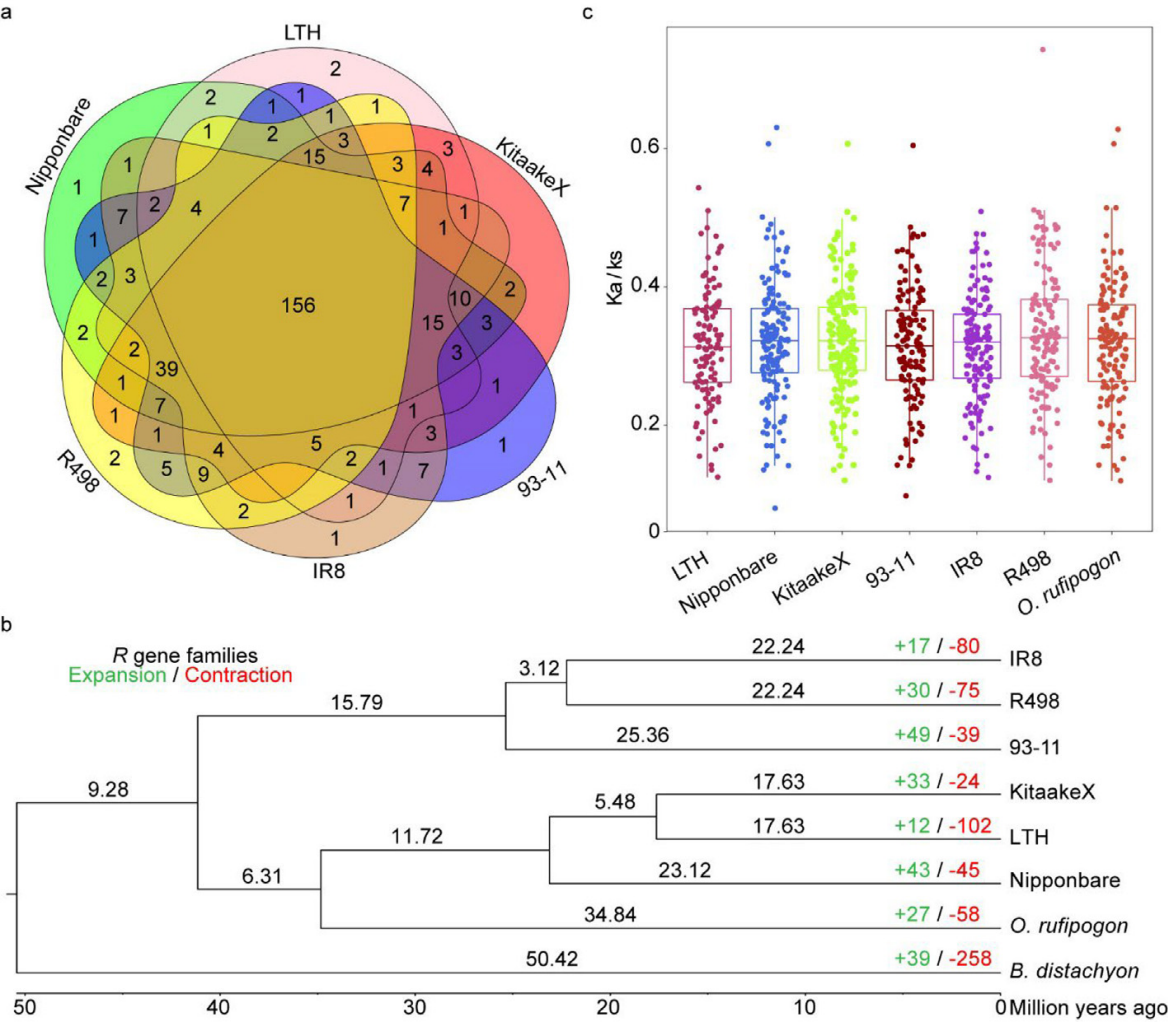
*R* genes analysis of Lijiangxintuanheigu (LTH) and other rice varieties. (a) The Venn diagram of *R* gene families in LTH, Nipponbare, KitaakeX, 93–11, Shuhui498 (R498), and IR8. (b) *R* gene family expansion and contraction analysis of seven rice varieties using *B. distachyon* as the outgroup. (c) The boxplot of Ka/Ks values of pairs of *R* genes between each rice variety and *B. distachyon.*

To further explore why the number of *R* genes in LTH is less than other rice varieties. We analyzed the relationship of *R* genes in rice varieties including Nipponbare, KitaakeX, 93–11, IR8 and R498, and the ancestor of rice *O. rufipogon* using *B. distachyon* as the outgroup. The 3,394 *R* genes were divided into 361 gene families, and most of the gene families are shared by the 6 rice varieties. The common *R* gene families account for 43.21% of the total gene families, and the rest are gene families only shared by some varieties (Fig. 5a). To further understand the expansion and contraction of *R* genes, we used café to further analyze the results above [77], and observed that LTH shows the lowest number of expansions but the highest number of contractions regarding *R* gene families among these rice varieties (Fig. 5b). This low level of expansion but high level of contraction may be the major reason for the small number of *R* genes in LTH. Meanwhile, we extracted the ortholog pairs of all *R* genes for selective pressure analysis in comparison to *B. distachyon*. We calculated the Ka/Ks of *R* genes in each rice variety and found that the median of Ka/Ks value in LTH is the lowest, but the values of Ka/Ks have no significance. Further analysis of the Ka/Ks values found that the ratio higher than 0.25 of Ka/Ks in LTH is the lowest in the *japonica* rice, suggest that the *R* genes in LTH may be under little selection pressure (Fig. 5c).

### 3.4 Selection pressure analysis in *japonica* rice varieties

**Fig. 6:**
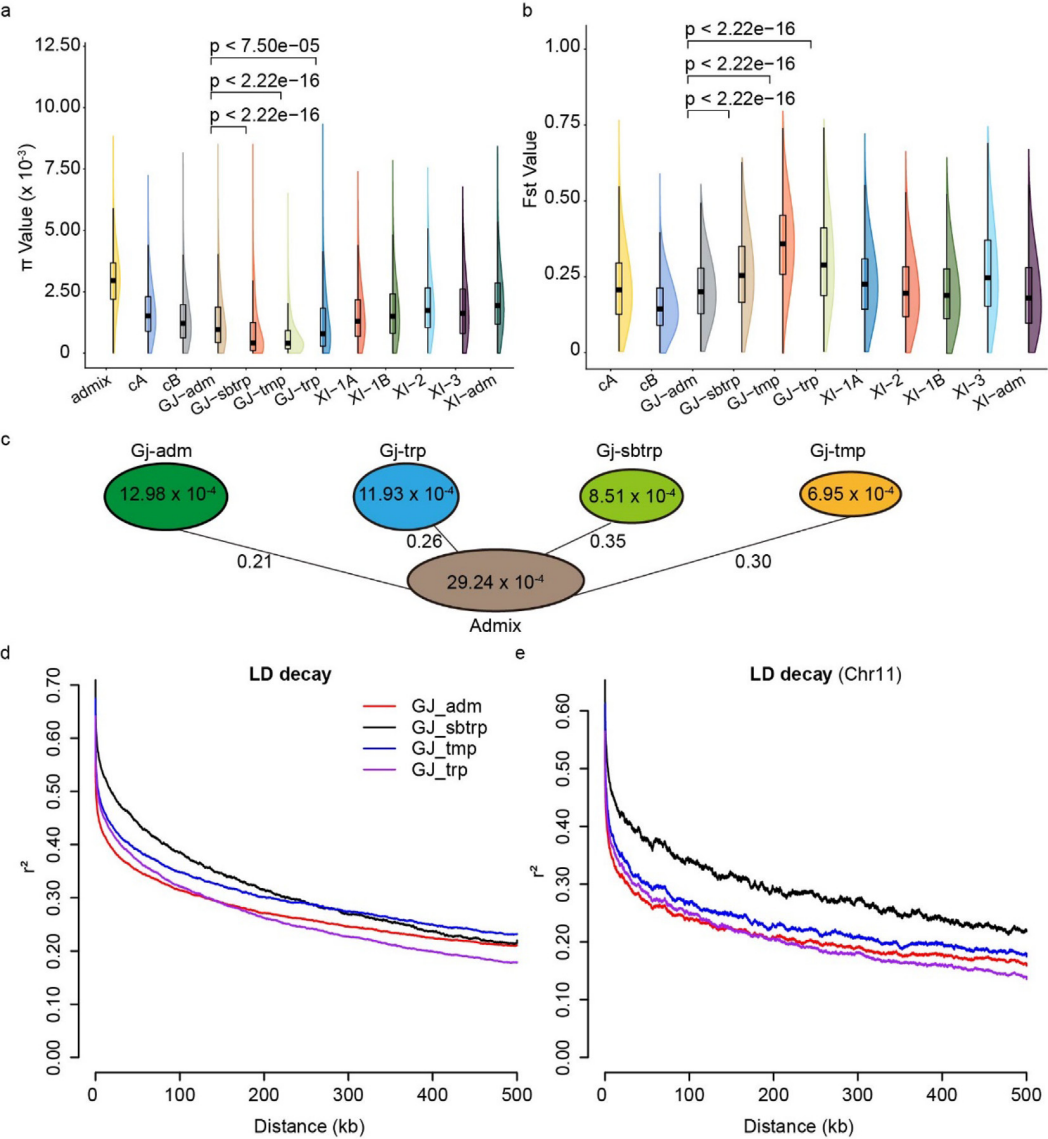
Genomic selection of the *japonica* populations. (a) and (b) Rain cloud plots of nucleotide diversity (π) and fixation index (Fst) for different rice groups, which include four XI clusters (XI-1A from East Asia, XI-1B of modern varieties of diverse origins, XI-2 from South Asia and XI-3 from Southeast Asia); three GJ clusters [primarily East Asian temperate (named GJ-tmp), Southeast Asian subtropical (named GJ-sbtrp) and Southeast Asian Tropical (named GJ-trp)]; and two groups for the mostly South Asian cA (circum-Aus) and cB (circum-Basmati) accessions. Accessions with admixture components < 0.65 within XI and GJ were classified as XI-adm and GJ-adm, respectively, and accessions that fell between major groups were classified as admixed. (c) Nucleotide diversity of the four *japonica* subpopulations and their Fst values for comparison with the Admix subpopulation. The value in each circle represents the nucleotide diversity of the subpopulation, while the value on each line represents the Fst value between indicated subpopulations. (d) and (e) The LD decay plots of the whole genome and chromosome 11.

To further explore the selection pressure on LTH, we performed analyses on the 3 K rice dataset [26]. According to the K9 nomenclature in the 3 K rice dataset, LTH belongs to the Gj-adm subpopulation while Kitaake and Nipponbare belong to Gj-tmp [21]. Compared with the other three *japonica* subpopulations including Gj-sbtrp, Gj-tmp and Gj-trp, the nucleotide diversity (π) for Gj-adm (π = 12.98 × 10–4) is significantly higher than those of Gj-sbtrp (π = 8.51 × 10–4, P < 2.22e-16), Gj-tmp (π = 6.95 × 10–4, P < 2.22 e-16) and Gj-trp (π = 11.93 × 10–4, P < 7.50e-05) (Fig. 6a and Fig. 6c). Compared with the Admix subpopulations, we calculated the fixation index (Fst) value (Fig. 6b) for each *japonica* subpopulation and found that the Fst for Gj-adm is significantly lower than that for the other three *japonica* subpopulations (P < 2.22e-16). We also performed the linkage disequilibrium (LD) test in these subpopulations, and found that the LD decay to half of its maximum value in Gj-adm is shorter than the other three subpopulations (Fig. 6d). We further performed the LD test on chromosome 11 (Fig. 6e) that harbors the most *R* genes in the rice genome, and the same result was obtained. Taken these results together, the subpopulation to which LTH belongs possibly receives the lowest selection pressure in the *japonica* population. Our results are consistent with the previous reports that the *M. oryzae* population in Yunnan, China is a genetic pool [99] that continuously generates new genotypes to adapt to the host, and that the subpopulation containing LTH, local germplasm in Yunnan, has received a very low rice blast selection compared other rice varieties. Thus, we speculate that *R* genes may be lost in LTH due to the low pathogen selective pressure in its local environment and the high genetic variation rate for its subpopulation.

### 3.5 A smaller number of plant defense-related genes are induced by rice blast in LTH than in Kitaake

**Fig. 7:**
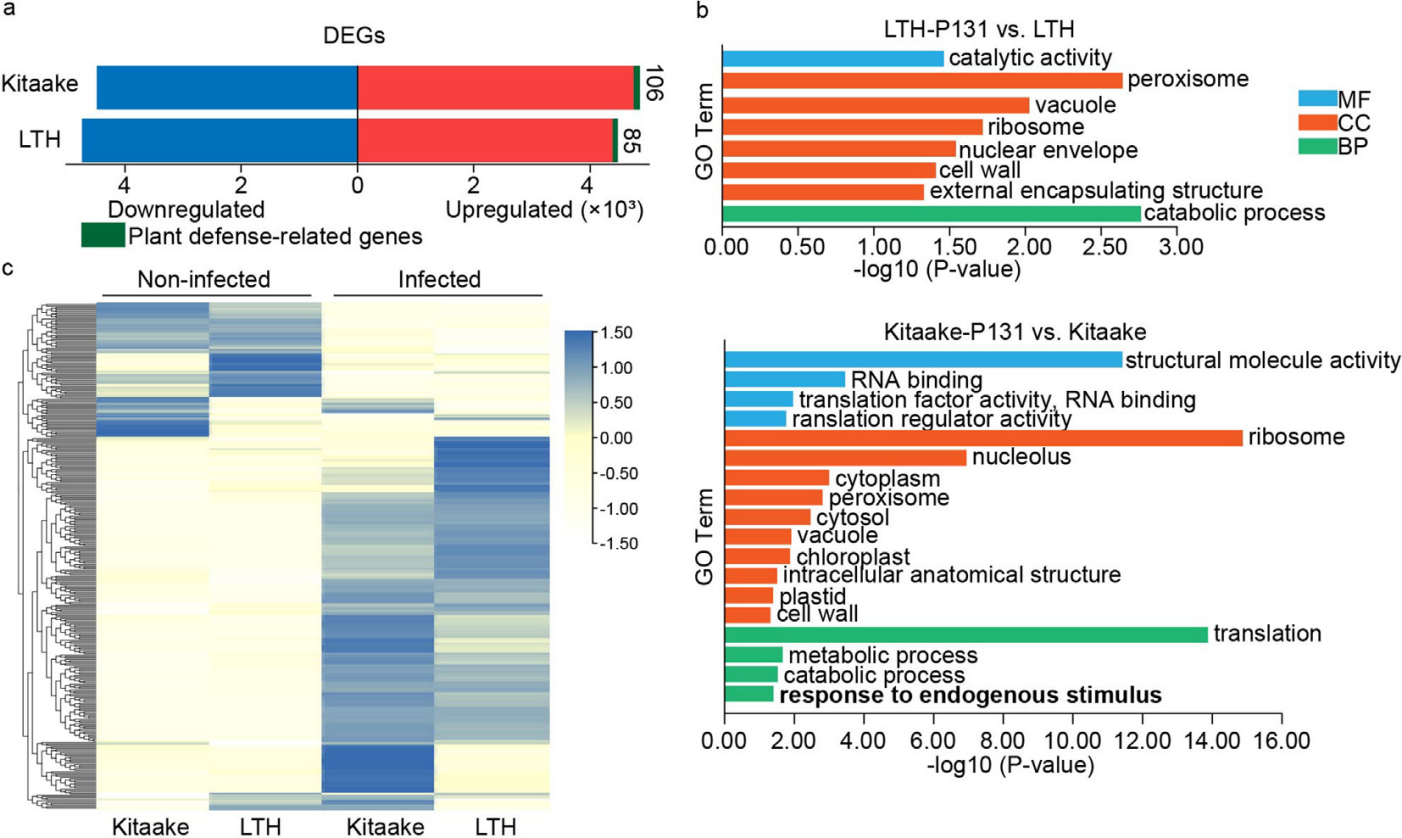
RNA-seq analysis of LTH and Kitaake infected by rice blast. (a) Differentially expressed genes (DEGs) between infected tissues with *M. oryzae* strain P131 and non-infected tissues of LTH and Kitaake. The top right numbers represent the numbers of plant defense-related genes. (b) The GO term enrichment analysis of upregulated DEGs in (a). (c) Heatmap visualization of expression levels of plant defense-related genes. The color key represents the normalized Z-score values of Transcripts Per Million (TPM).

We used transcriptomic assays to analyze the expression of plant defense-related genes in LTH when challenged with rice blast. The plant defense-related genes were retrieved from the funRiceGenes database that categorizes curated genes according to their published functions [34]. There are 85 plant defense-related genes upregulated in LTH under infection conditions (Fig. 7a). However, further analysis of all upregulated genes showed that there is no enriched GO term related to immunity identified (Fig. 7b). In contrast, RNA-seq analysis of Kitaake under the same condition revealed that there are 106 upregulated plant defense-related genes compared with LTH, and the enriched GO term named “response to endogenous stimulus” was identified for upregulated genes (Fig. 7b), indicating that a significant number of DEGs might be involved in response to rice blast in Kitaake. We also found that the gene expression was induced to a higher level for most plant defense-related genes in Kitaake than that in LTH, though the genes were induced in both varieties (Fig. 7c).

### 3.6 Pattern-triggered immunity in LTH is normal

**Fig. 8:**
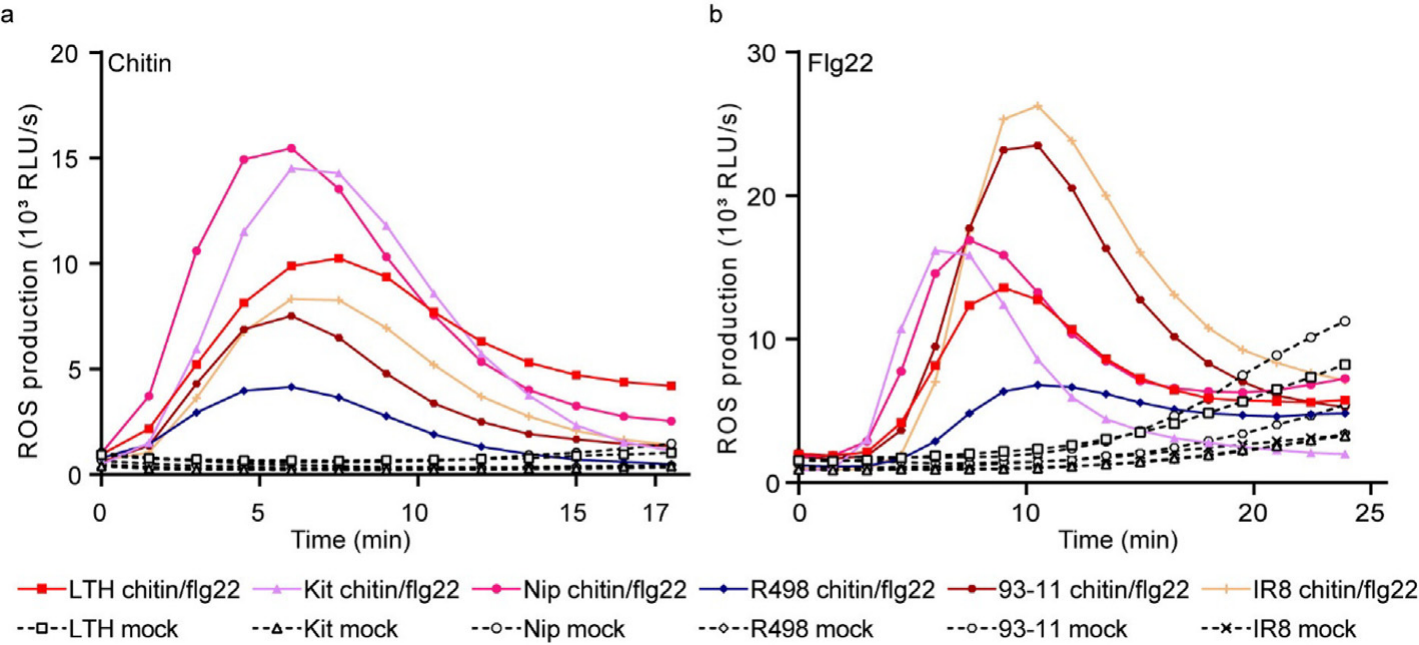
ROS accumulation dynamics in Lijiangxintuanheigu (LTH) and other rice varieties challenged with chitin and flagellin22 (Flg22), respectively. Other rice varieties include Nipponbare (NIP), Kitaake (Kit), IR8, 93–11, and Shuhui498 (R498). Water was used for the mock treatment.

PTI is an important layer of the plant immune system, regulating basic defense reaction against a wide range of pathogens. We analyzed core proteins involved in PTI in LTH, including CERK1, CEBiP, FLS2, and Rac1, and observed that these proteins, with the exception of FLS2, are identical to the functional ones. The FLS2 in LTH lacks nine amino acids at its N-terminus as compared to, cloned FLS2 (Fig. S9). We further tested the function of the PTI system by analyzing the ROS generated by LTH challenged with the PAMPs chitin and flagellin22. Chitin is a typical component of the rice blast cell wall, and flagellin22 is the 22-amino acid conserved fragment of the bacterial flagellin, and both induce ROS bursts as part of PTI. During the chitin challenge, ROS levels in the LTH are a little lower in LTH than Nipponbare or Kitaake, but higher than IR8, 93–11, and R498 (Fig. 8a and b). Similar results were observed for the flagellin22 treatment. These results demonstrate that after rice blast infected, PTI is likely normal in LTH, in contrast to the weak ETI mediated primarily by *Pi* genes.

### 3.7 Rapid cloning of the *R* gene *Piak* from Kitaake facilitated by the LTH genome

**Fig. 9:**
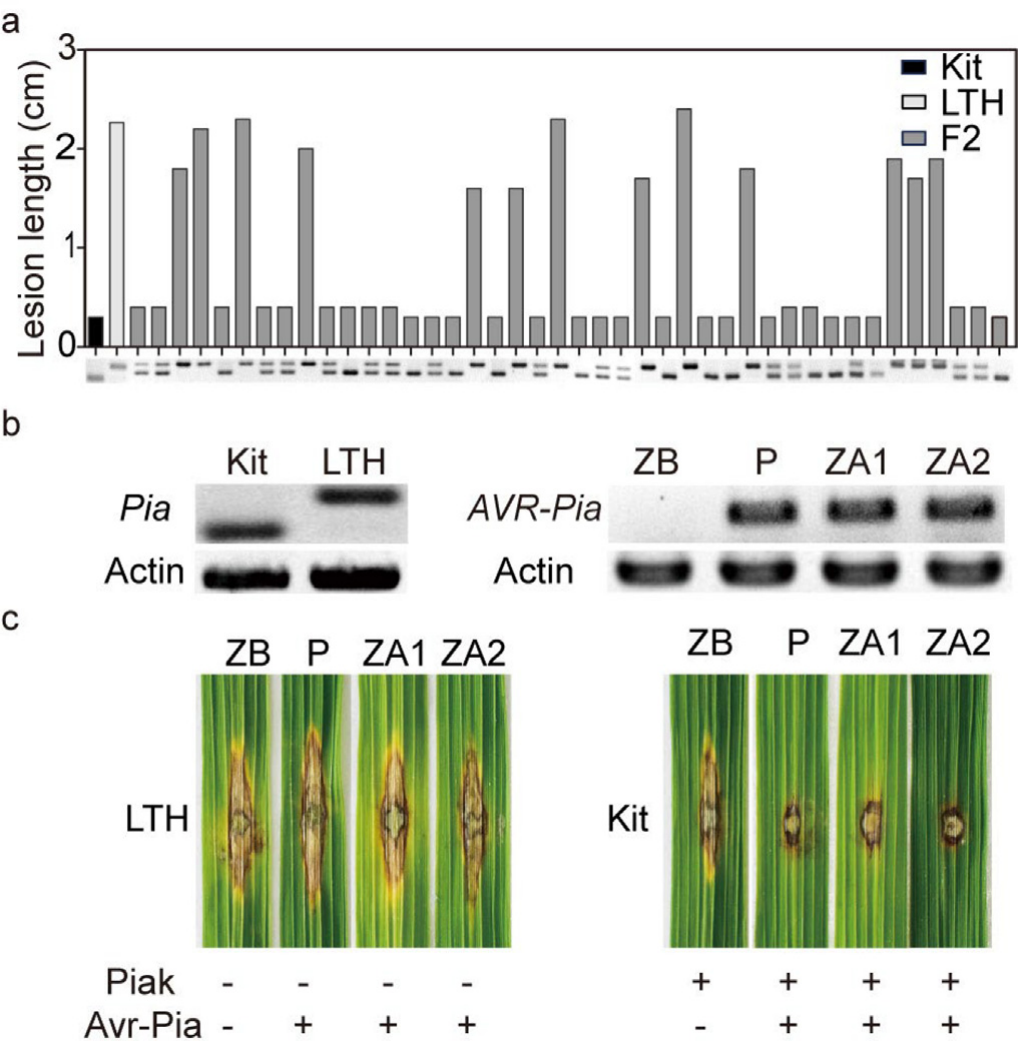
Rapid cloning of *Piak* from Kitaake (Kit). (a) The *Pia* locus co-segregates with the resistance phenotype in the F2 population derived from Kitaake crossed with Lijiangxintuanheigu (LTH). In genotyping, a small band indicates a functional *Pia* allele, and a large band a nonfunctional one. (b) Genotyping of the *Pia* locus in rice Kit and LTH as well as the *AVR-Pia* gene in *M. oryzae* strains ZB25 (ZB), P131 (P), and ZB25 carrying *AVR-Pia* (ZA1, ZA2). The amplicon of the actin gene was used as the control. (c) Interaction between *Pia* and *Avr-Pia* confers Kit resistance to strains P, ZA1 and ZA2. “+” and “-” indicate the presence and absence of indicated proteins, respectively.

Rice variety Kitaake displays moderate resistance to *M. oryzae*, including the field isolate P131 [30]. To clone the *R* gene against P131, we crossed Kitaake with LTH to obtain the segregating F2 population, in which the ratio of resistant plants to susceptible plants is 961:278. A χ^2^ test of the phenotypic ratio revealed that the actual value is statistically similar to the expected value (3:1, χ^2^ = 2.238, P-value = 0.131 > 0.05), and the resistance phenotype is controlled by a dominant locus. We took advantage of the availability of Kitaake and LTH genomes and identified putative *Pi* genes from Kitaake and related genetic markers in comparison to LTH. In co-segregation assays, we observed that the *Pia* locus rather than *Pish*, *Pi-cd* or *Pi37* co-segregated with the resistance phenotype in the segregating F2 population (Fig. 9a, S10-12). We analyzed the F2 population and in 970 F2 plants the resistance co-segregated well with the *Pia* locus (Supporting data 5). We compared the *Pia* locus, comprising two paired *R* genes *RGA4* and *RGA5*, of Kitaake named as *Piak* to LTH in detail and observed that *RGA5* of the *Pia* locus is absent in LTH (Fig. S13), resulting in a nonfunctional *Pia* locus in LTH. *RGA5* in Kitaake encodes a protein 8 amino acids longer than the cloned one from the rice variety Sasanishiki [100] (Fig. S13).

To further verify the co-segregation results, we identified the *AVR-Pia* gene from the *M. oryzae* strain P131 but not ZB25 that is virulent to Kitaake (Fig. 9b). We transformed *AVR-Pia* from P131 into ZB25, and the resultant strains ZA1 and ZA2 became avirulent to Kitaake (Fig. 9c), confirming that the incompatible reaction between Kitaake and strains ZA1, ZA2 and P131 is controlled by the canonical Pia-Avr-Pia pair and that the *Piak* gene is responsible for the resistance to P131 in Kitaake.

## 4 Discussion

Using the Nanopore long reads together with the NGS short reads, we have assembled a high-quality reference genome for the rice variety LTH with a contig N50 of 22.7 Mb, which reaches the “gold standard” reference genome for rice [62]. As the sequencing technology advances, Nanopore sequencing data are robust enough in assembling a high-quality genome with correction using the NGS data for many organisms [101]. In the Basmati 334 rice genome, using Nanopore long reads, Choi et al. assembled the Basmati 334 genome to the chromosome level [88]. For the fungus *Peltaster fructicola*, the Nanopore long reads are sufficient to assemble a complete genome [102]. LTH has already been used to clone>10 *Pi* genes and other *R* genes. By comparing the LTH genome with other common rice varieties, we have further provided millions of genetic markers, a rich resource facilitating cloning of genes and other aspects of rice research. In traditional genetic mapping approaches, a large number of genetic markers are used to fine map the target gene, which usually takes a long time and related resources. With the availability of the LTH genome, researchers can accelerate this process as demonstrated by the rapid cloning of the *Piak* gene, which is consistent with the previous finding that Kitaake harbors a functional *Pia* locus [7] (Fig. 9).

LTH is universally susceptible to worldwide *M. oryzae* isolates [8]. In agreement with this phenotype, we observed that all of the 43 functional *Pi* genes including alternate alleles are either absent or lose functions in LTH due to genomic variations (Fig. 4). Unexpectedly, there are three predicted functional *R* genes that are allelic to *Pikm2*, *Pik-2* and *Piks-2*, which are in gene pairs [97], [98]. Gene clustering or pairing is rare in eukaryotes but *R* gene clustering or pairing is not uncommon in plants [113]. *R* gene clustering or pairing might contribute to the broad-spectrum resistance that most plants have with their finite number of *R* genes in their genomes. Paired genes are usually arranged in a head-to-head pattern, and the two genes have to work together and may represent the integrated decoy model for effector recognition [103]. Therefore, the predicted functional *Pikm2, Pik-2 and Piks-2* are likely nullified by the mutated working pairs of *Pikm1, Pik-1 and Piks-1*. The same situation happens to other paired genes in different rice varieties [100].

In addition to lacking of *Pi* genes, the number of *R* genes of LTH is the lowest among commonly used rice varieties (Fig. 3a). The low number of *R* genes is supported by the low level of *R* gene family expansions and high level of *R* gene family contractions, which might also contribute the susceptible immune response of LTH to rice blast (Fig. 5b). Consistent with our results, by analyzing>90,000 *R* genes from over 300 angiosperm genomes [104], Liu et al. indicate that the number of *R* genes in plants is broadly associated with pathogen resistance, as viewed across evolutionary lineages. It should be mentioned that the number of *R* genes is not always directly proportional to disease resistance due to differences in the genome size and complexity [23], [105]. Surprisingly, we observed that the number of LRR-RLKs in LTH is significantly reduced (Fig. 3a). RLKs, particularly LRR-RLKs are important in plant immunity [106]. For example, FLS2 and the EF-Tu receptor (EFR) in Arabidopsis are well characterized LRR-RLKs in plant defense [107]. In LTH, the atypical *R* gene Pi-d2, a RLK, and *bsr-d1/k1* are nonfunctional due to alterations in the amino acid residues or the nucleotide in the promoter [9] (Figs. S14 and S15), which might also contribute to the susceptibility of LTH. Yet, there are still 338 *R* genes in LTH, any of which could be functional against rice blast. However, we show that the median Ka/Ks value of available *R* genes in LTH is the lowest among commonly used rice varieties, and the Ka/KS ratio above 0.25 of LTH is also the lowest in the three *japonica* rice varieties. Additionally, we found the lowest selection pressure and the highest variation ratio in the subpopulation to which LTH belongs in the 3 K rice data. These results together indicate that the *R* genes in LTH may be under weak selection pressure of the dynamic rice blast pathogen population. In other words, *R* genes in LTH may be less likely functional or function at the minimal level in avoiding the pathogen pressure (Fig. 5c), which may be a reason for the universal susceptibility of LTH to rice blast. The value of Ka/Ks of the different rice varieties concentrate on 0.2–0.4, indicating that most *R* genes tend to be purified in rice. Similar scenarios are observed in leguminous plants [108], which may limit the evolution of *R* genes. In addition, the ratio of Ka/Ks>0.25 in LTH is the smallest among the three *japonica* rice varieties, which indicates that more *R* genes in Nipponbare and KitaakeX may be positively selected, making them more resistant to diseases than LTH. Population genetics analyses also demonstrate that the subpopulation to which Kitaake and Nipponbare belong has the higher selection pressure than LTH faces. In addition, the gene expression analysis shows that many more plant defense-related genes are upregulated in Kitaake compared to LTH under rice blast conditions. We also hypothesize that the LTH-containing plant defense-related genes are possibly rarely selected and functional against other pathogens, too. In supporting the hypothesis, LTH is shown to be highly susceptible to the rice bacterial blight pathogen *Xanthomonas oryzae* pv. *oryzae* (*Xoo*) [11]. In an assay, LTH is susceptible to all of the six *Xoo* pathotypes with varied virulence collected in South China, indicating that LTH can be used as the susceptible control in cloning *R* genes against *Xoo*.

PTI is usually responsible for the initial defense response to microbial pathogens [3]. The level of PTI in LTH is moderate compared to other rice varieties (Fig. 8), unlike the attenuated ETI mediated primarily by *Pi* genes in LTH. This observation is consistent with the recent reports that plant ETI and PTI are linked and that effective plant defense against pathogens relies on both effective PTI and ETI systems [4], [5]. When ETI functions at a low level, it is likely that a similar situation occurs for PTI. Therefore, we propose that the low level of ETI mediated primarily by *Pi* genes restricts the ability of LTH to trigger effective PTI. Overall, the universal susceptibility of LTH to thousands of worldwide *M. oryzae* isolates [109] primarily results from the attenuated ETI system primarily mediated by *Pi* genes.

The immune system of LTH is not completely disrupted and could be partially functional. For example, LTH has been widely used as the recipient in cloning *R* genes, and transfer a *Pi* gene to LTH is sufficient to confer LTH resistance to certain *M. oryzae* isolates as demonstrated in the differential varieties [110] and in our study (Fig. 9). Furthermore, mutation of one site is sufficient to confer LTH broad-spectrum resistance to multiple *Xoo* strains [11]. In addition, RNA-seq assays show that there are plant defense-related genes expressed at low levels in LTH (Fig. 7). These examples demonstrate that the immune system of LTH is severely attenuated but could be functional.

In conclusion, with extensive analyses of plant immunity-related genes in the newly assembled LTH genome and experimental assays, we show that the universal susceptibility of LTH to rice blast is not because its PTI system, but the attenuated ETI system to rice blast. Attenuated ETI of LTH is primarily because it lacks functional *Pi* genes, and is possibly associated with reduced numbers of *R* genes and LRR-RLKs as well as minimally functional or nonfunctional plant defense-related genes available in the genome. We also assessed the heterozygosity of SNPs in the LTH genome in the 3 K rice data, and the heterozygosity of SNPs in the LTH genome is 3.8%, which means that LTH is an ideal line for genome-wide association analysis and *R* gene cloning. The high-quality reference genome of LTH will significantly accelerate *Pi* gene cloning and other aspects of rice functional genomics studies.

## Declaration of Competing Interest

The authors declare that they have no known competing financial interests or personal relationships that could have appeared to influence the work reported in this paper.
