# Supplementary material for: The genome of the rice variety LTH provides insight into its universal susceptibility mechanism to worldwide rice blast fungal strains
Source: Comput Struct Biotechnol J. 2022 Feb 10;20:1012–26. doi: 10.1016/j.csbj.2022.01.030 (PMC8866493; doi:10.1016/j.csbj.2022.01.030)

**Figure S1** LTH in the MDS plot of 3010 rice accessions


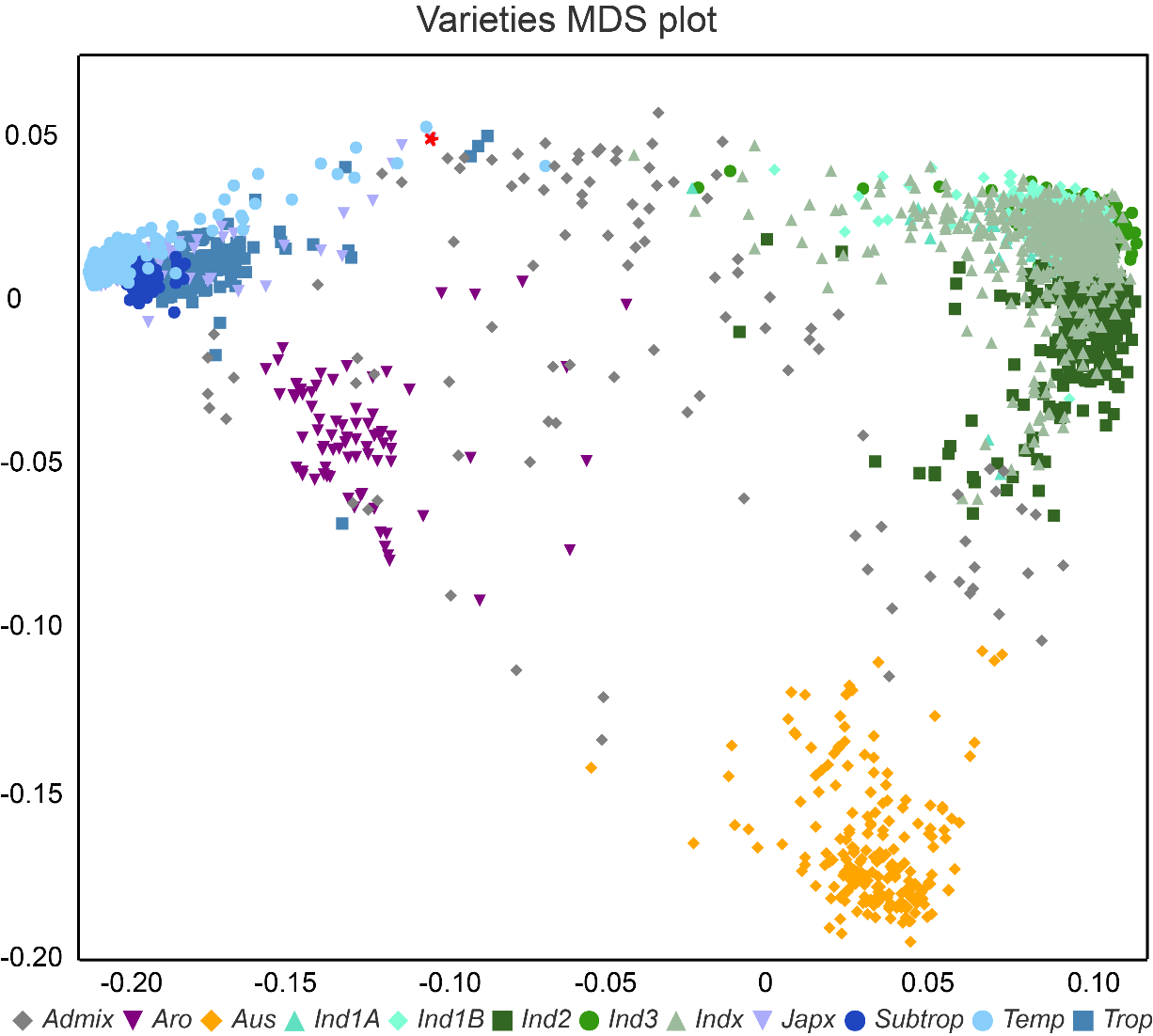


The MDS plot shows the relationships between the 12 subgroups. These subgroups are mainly related to geographic location. There were four Ind subgroups (Ind1A from East Asia, Ind1B of modern varieties of diverse origins, Ind2 from South Asia and Ind3 from Southeast Asia); four Geng/Japonica subgroups (primarily East Asian temperate (named tmp), Southeast Asian subtropical (named sbtrp) and Southeast Asian tropical (named trp), the Japx was included in these); two groups for the mostly South Asian (named Aus and Aro), admix is admixed between any two or more of the above groups.

**Figure S2** The *k*-mer analysis of the LTH genome


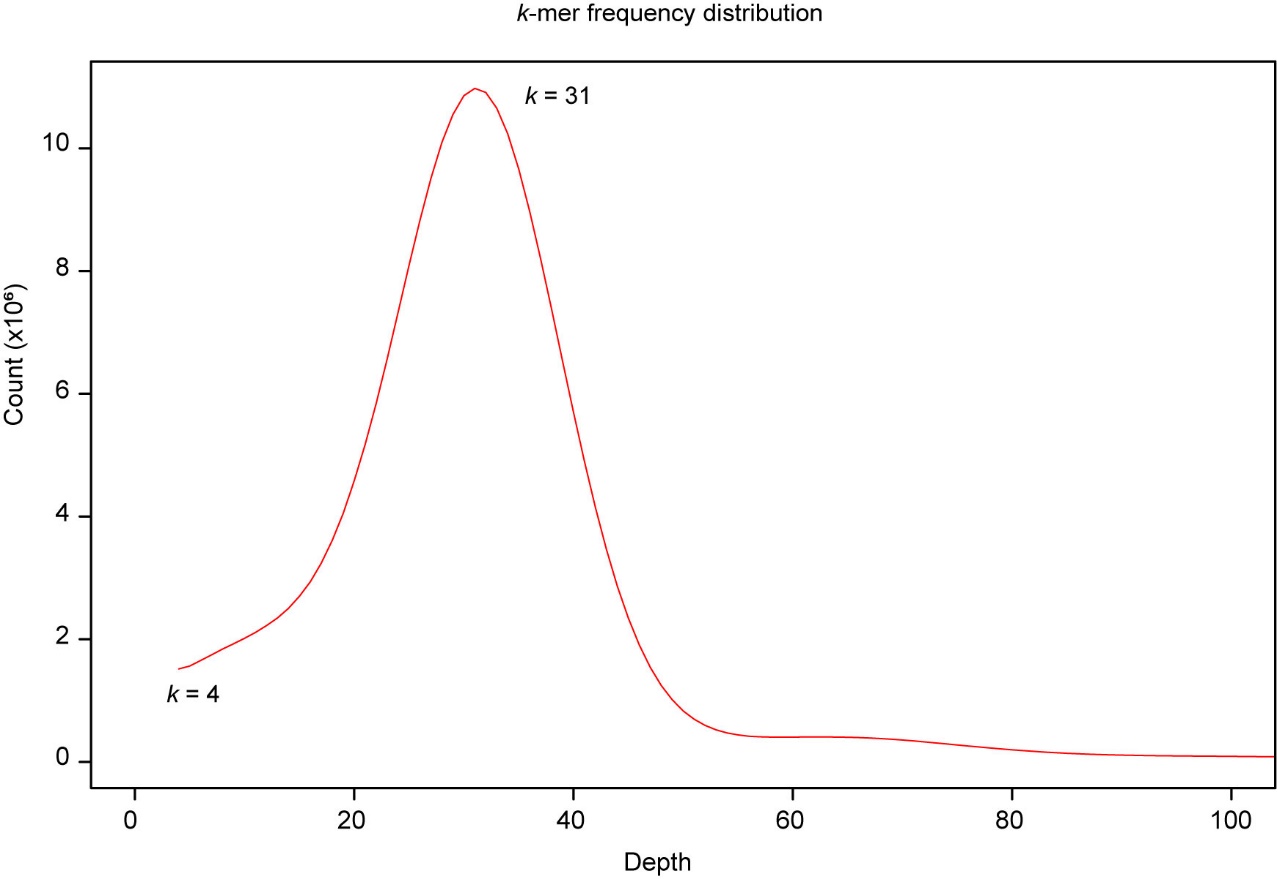


**Figure S3** Genome-wide distribution of the short reads in the LTH genome


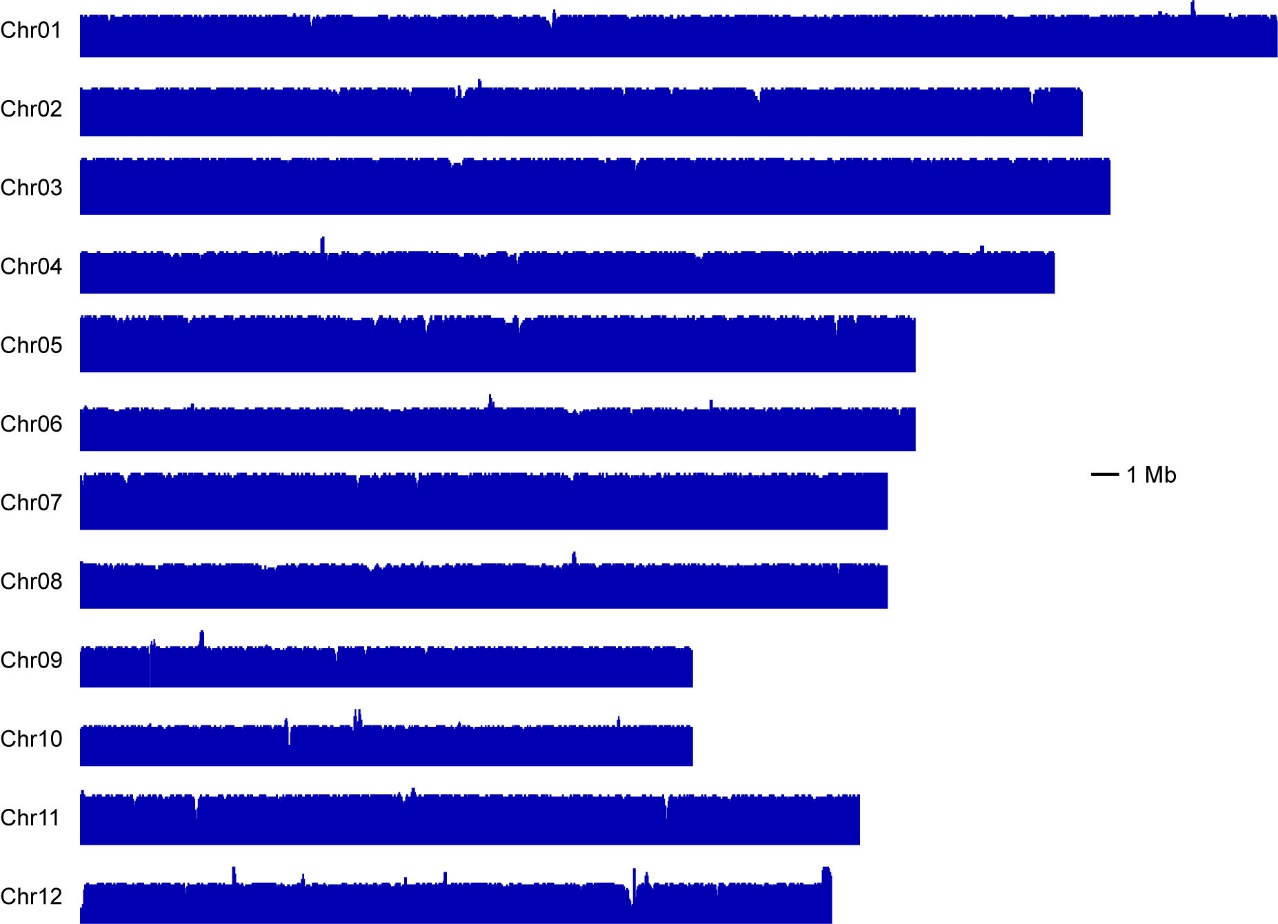


**Figure S4** Dot plot comparing the LTH and Nipponbare genomes


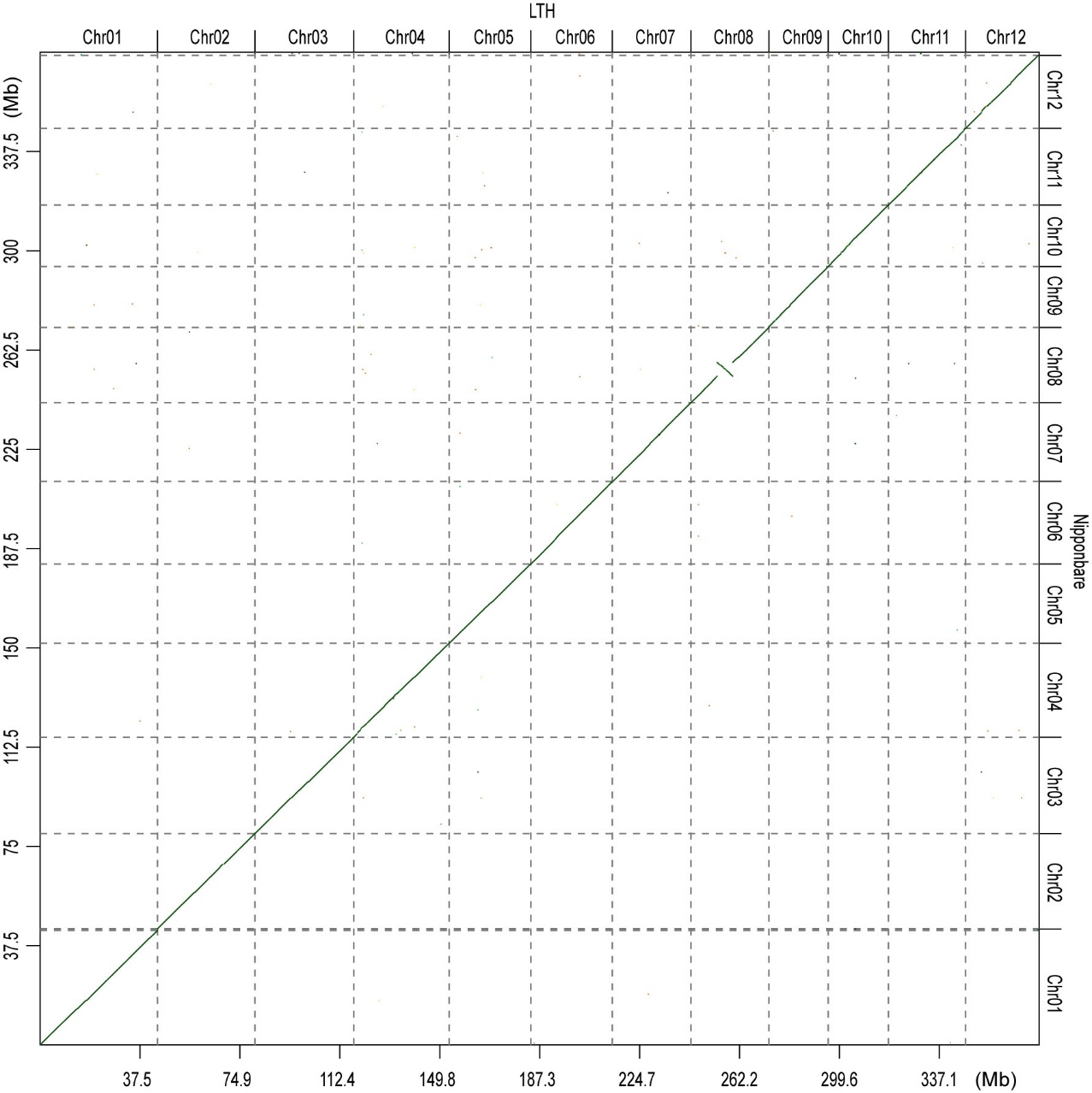


**Figure S5** The functional effect of SNPs between KitaakeX and Nipponbare


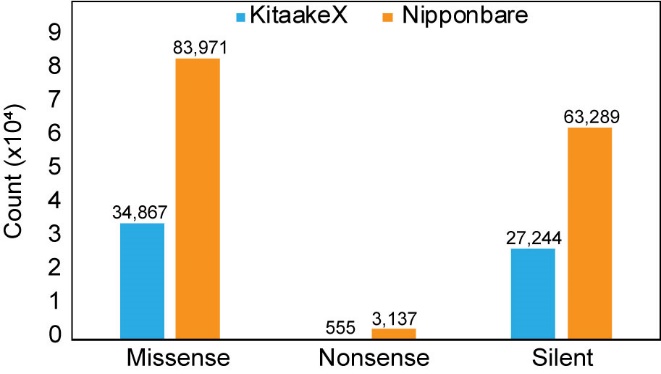


**Figure S6** The effect classification of the variations between KitaakeX and Nipponbare


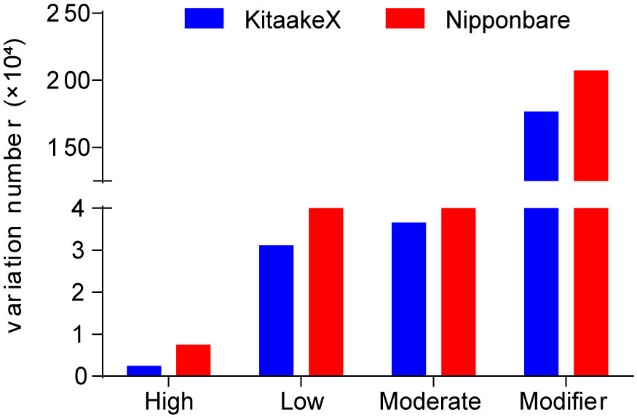


**Figure S7** Genome-wide distribution of *R* genes in rice varieties LTH and KitaakeX


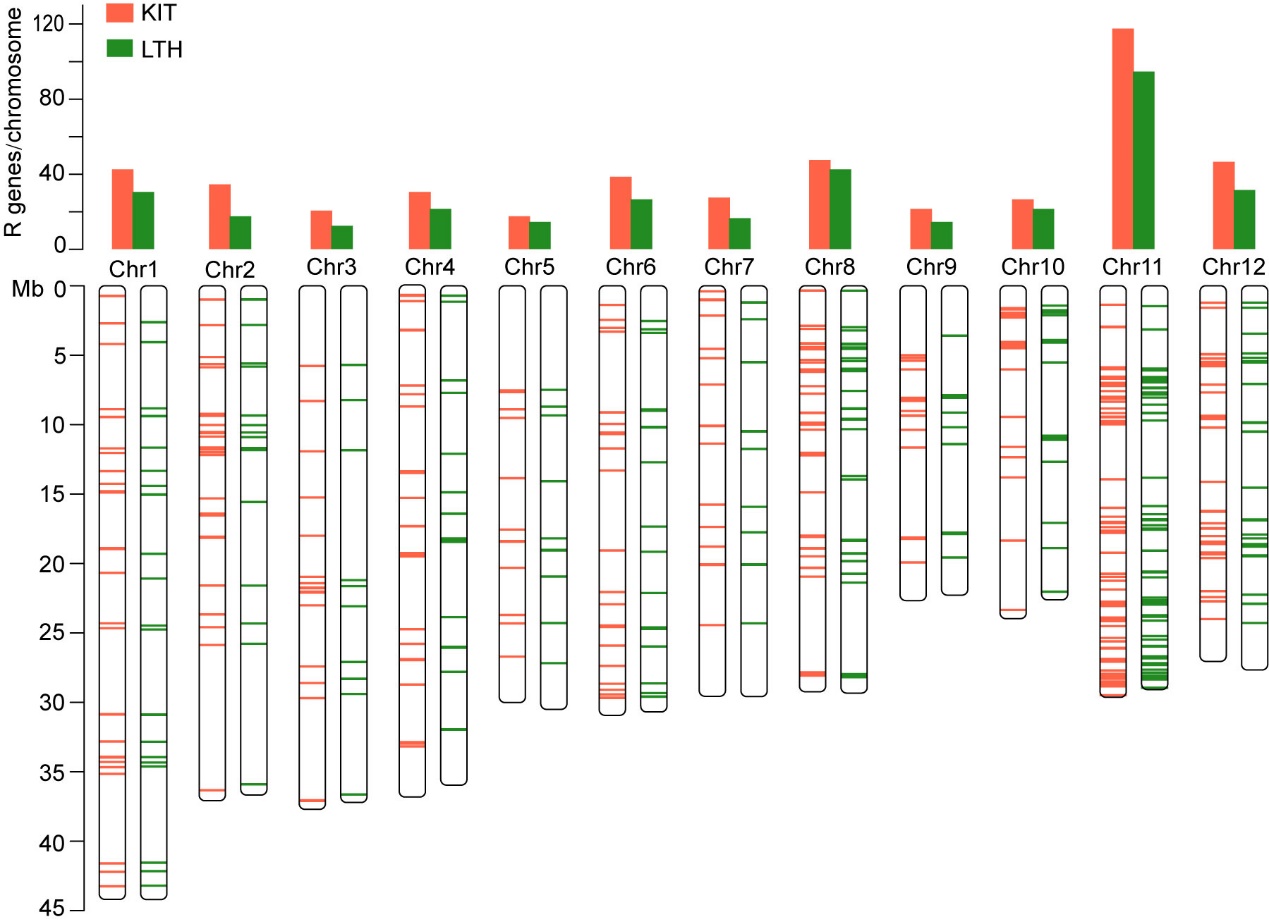


**Figure S8** The alignment of *Pi* genes in LTH genome


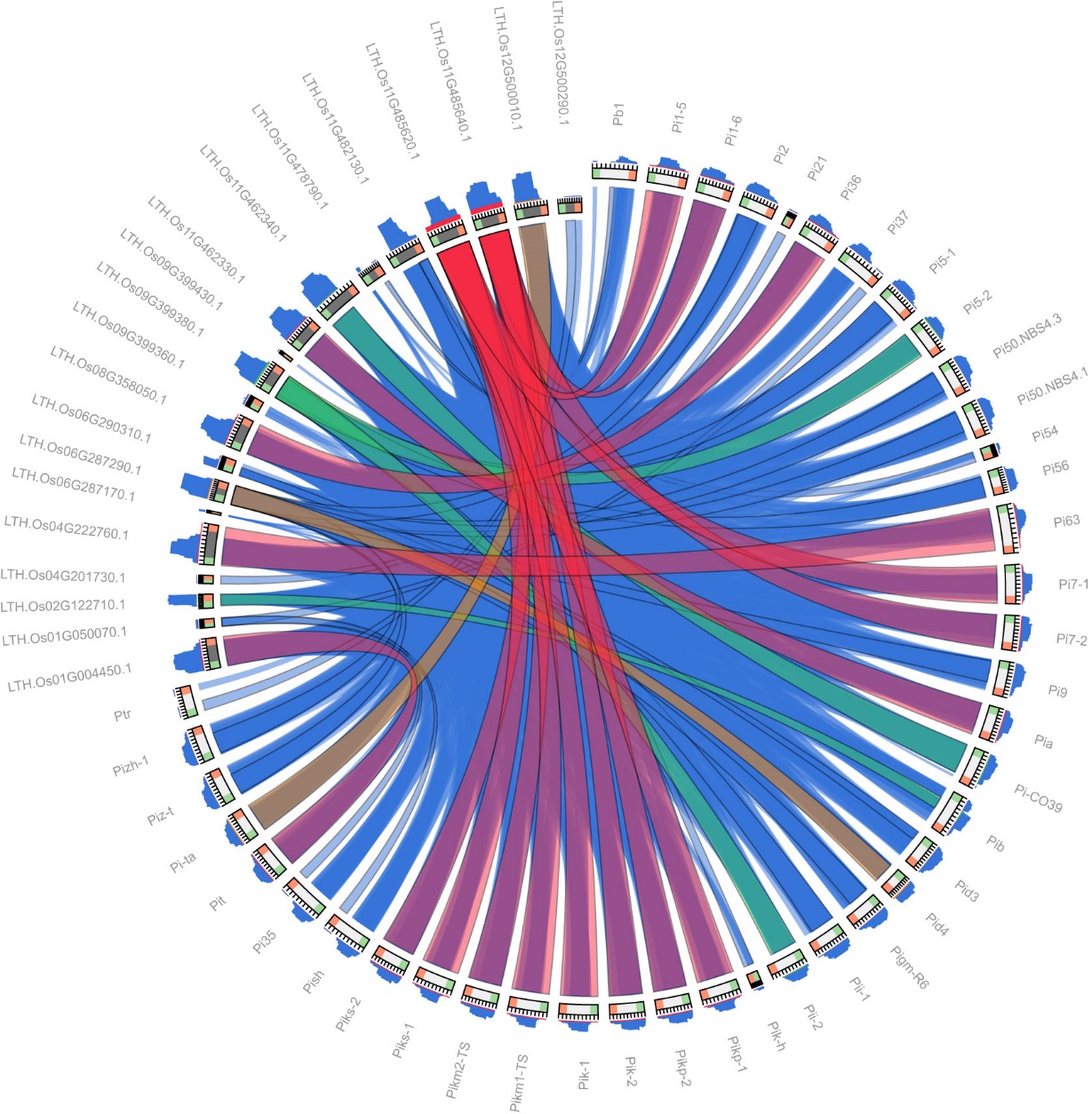


Comparison of different *Pi* genes in the LTH genome, the ribbons were colored using the (score-min score) / (max score-min score) ratio with blue<=0.25, green<=0.50, orange<=0.75, red>0.75. The histograms indicate the blast scores.

**Figure S9** Alignment of the candidate LTH gens to FLS2


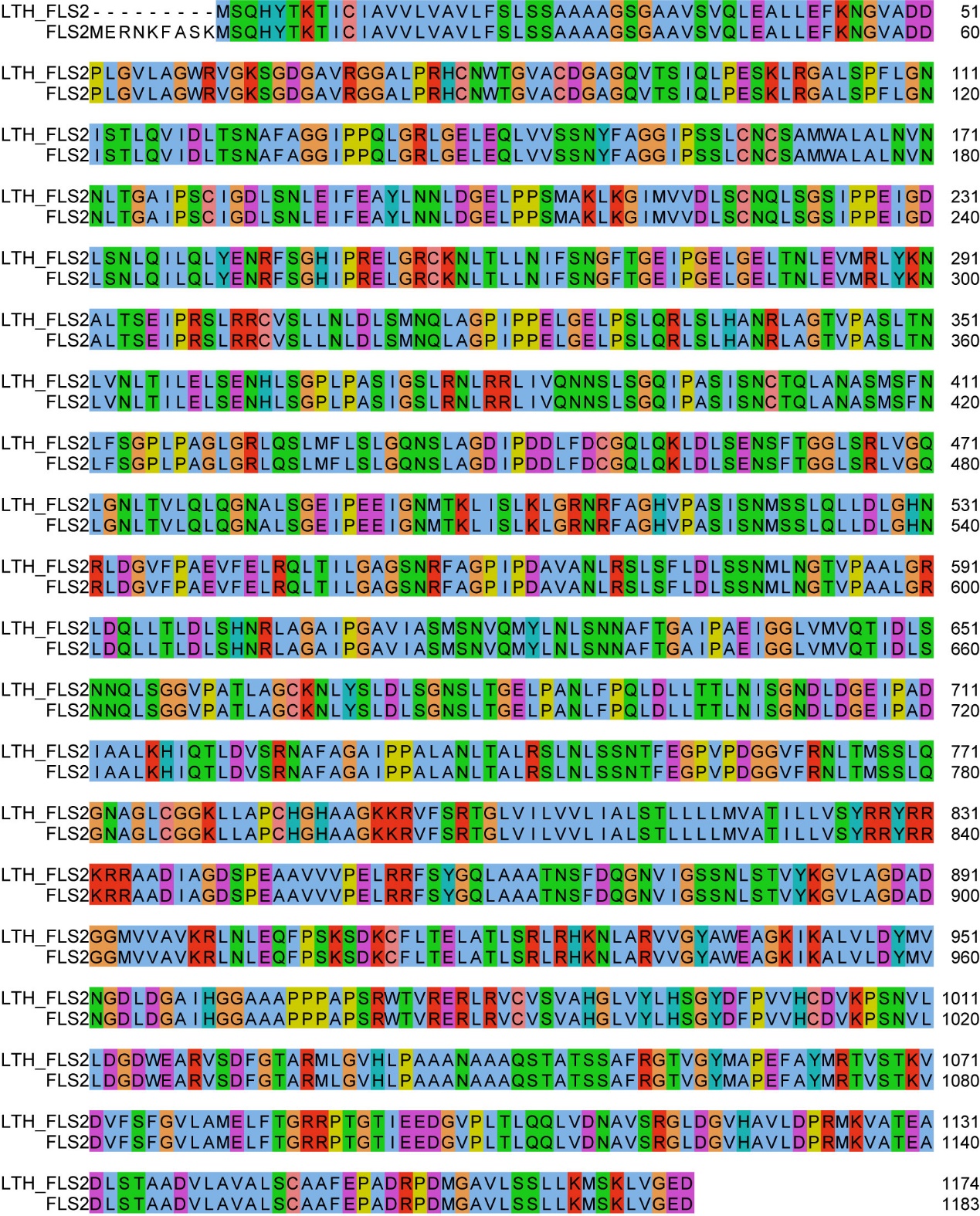


**Figure S10** The *Pish* locus does not co-segregate with the resistance phenotype


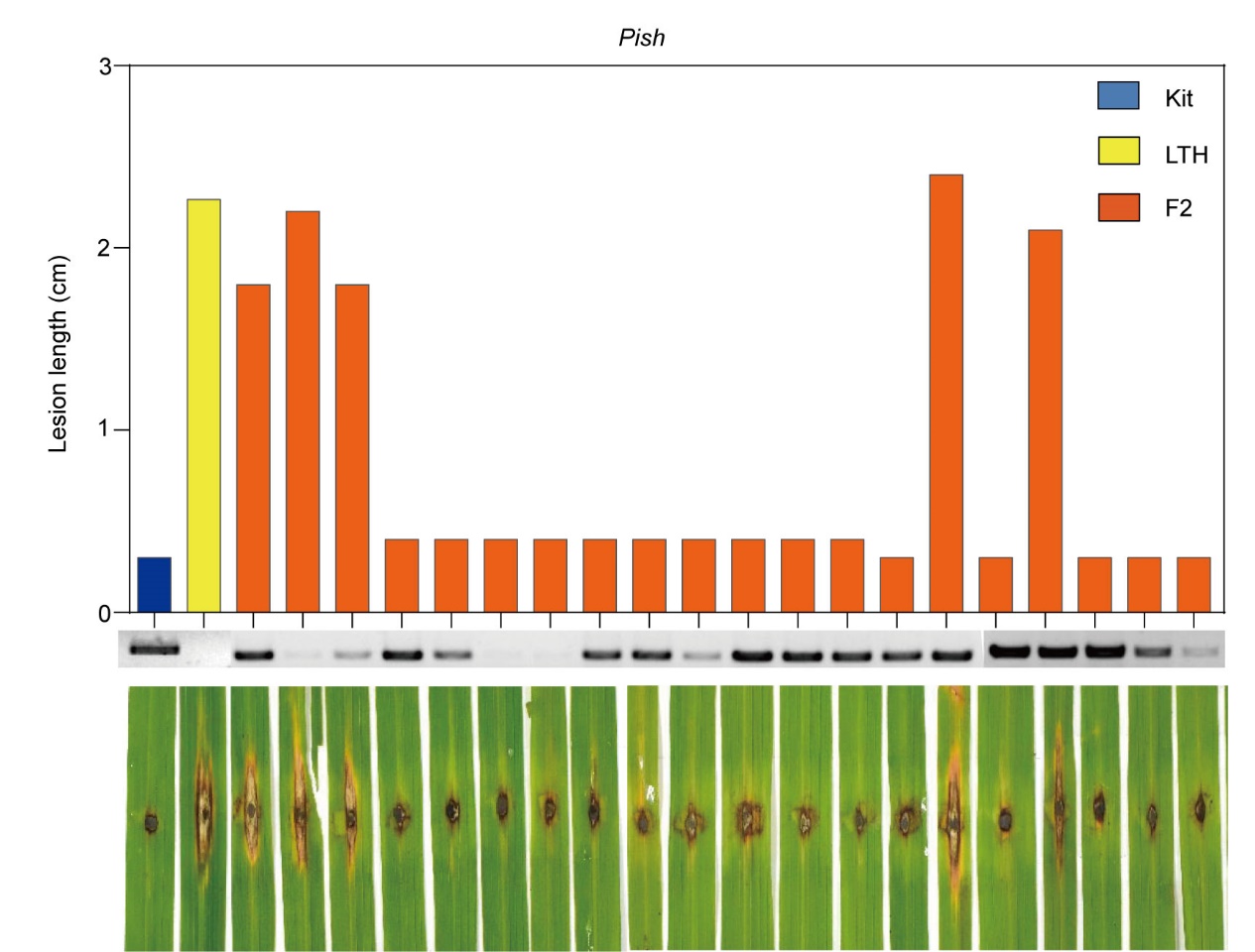


**Figure S11** The *Pi-cd* locus does not co-segregate with the resistance phenotype


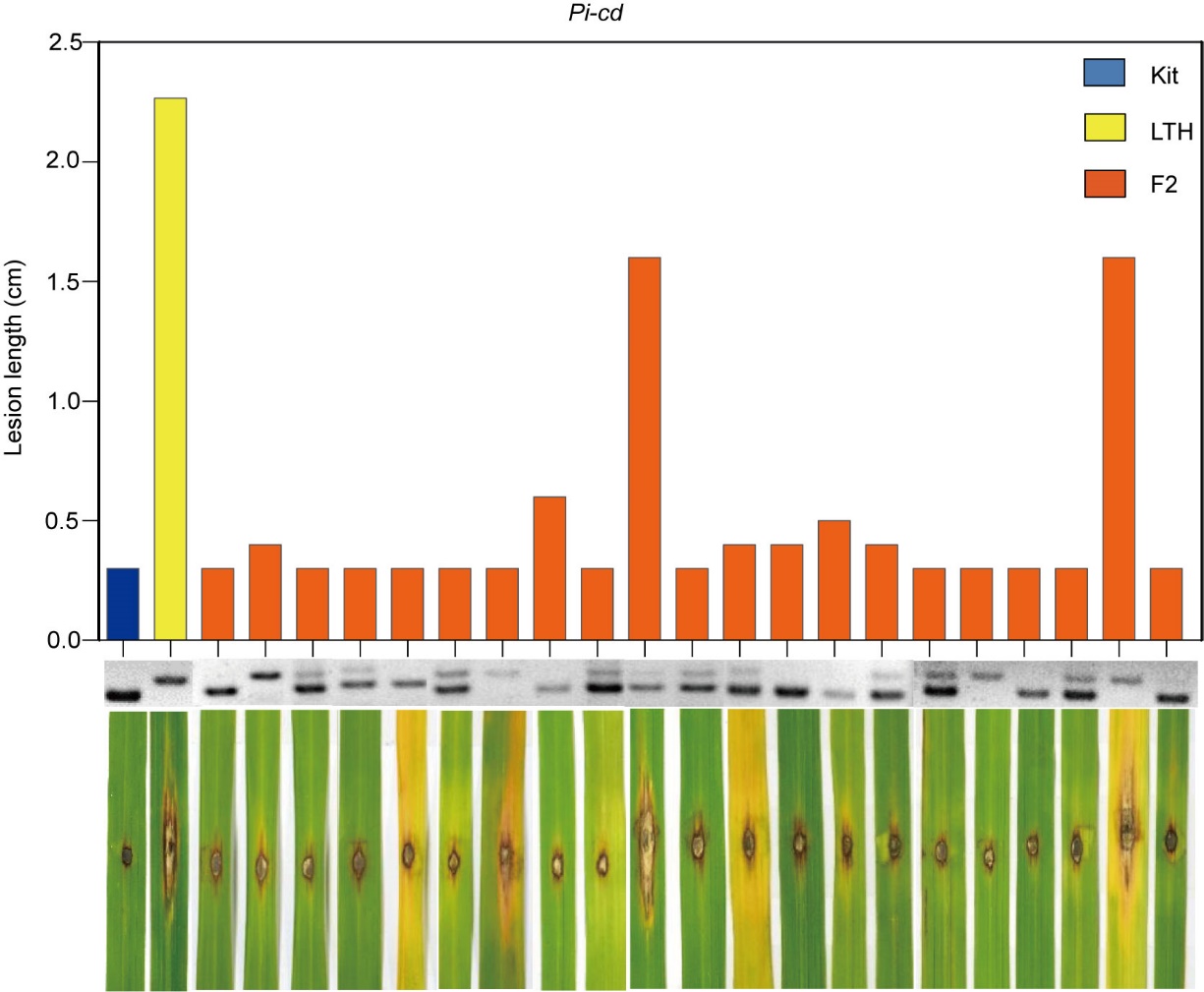


**Figure S12** The *Pi37* locus does not co-segregate with the resistance phenotype


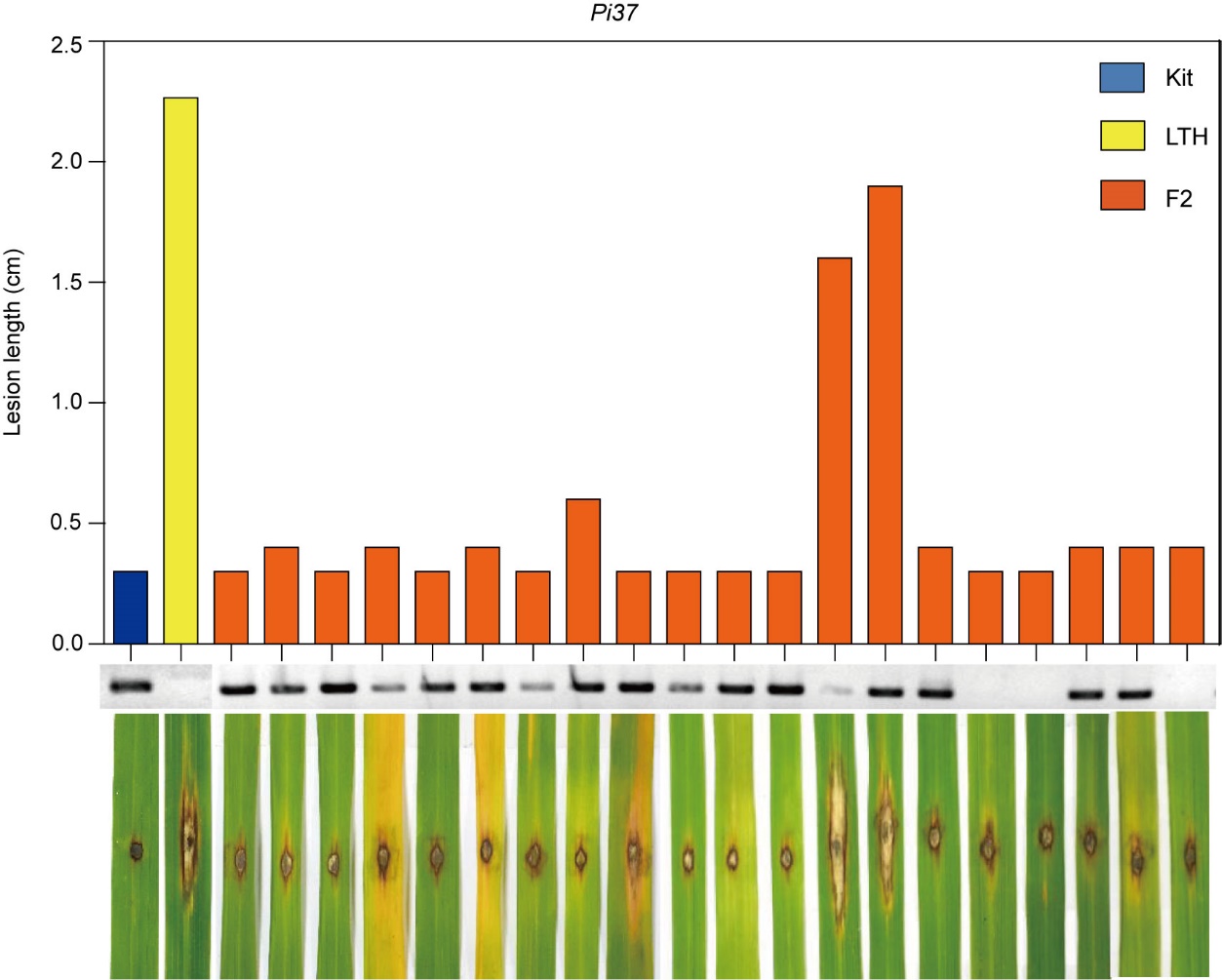


**Figure S13** Alignment of the functional allele RGA5 (Pi-CO39) to the candidate LTH allele


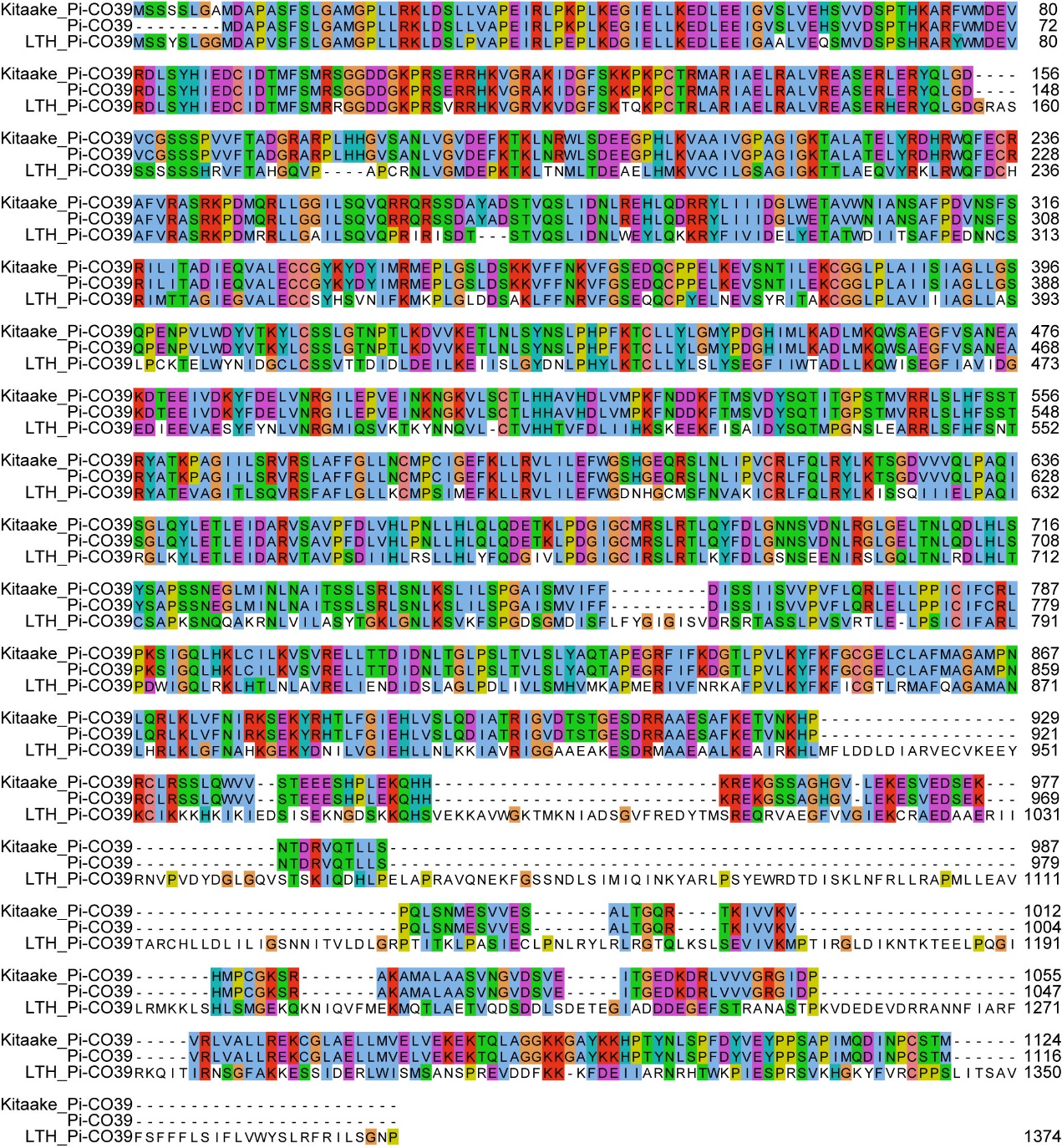


**Figure S14** Alignment of the candidate LTH and Kitaake genes to Pi-d2


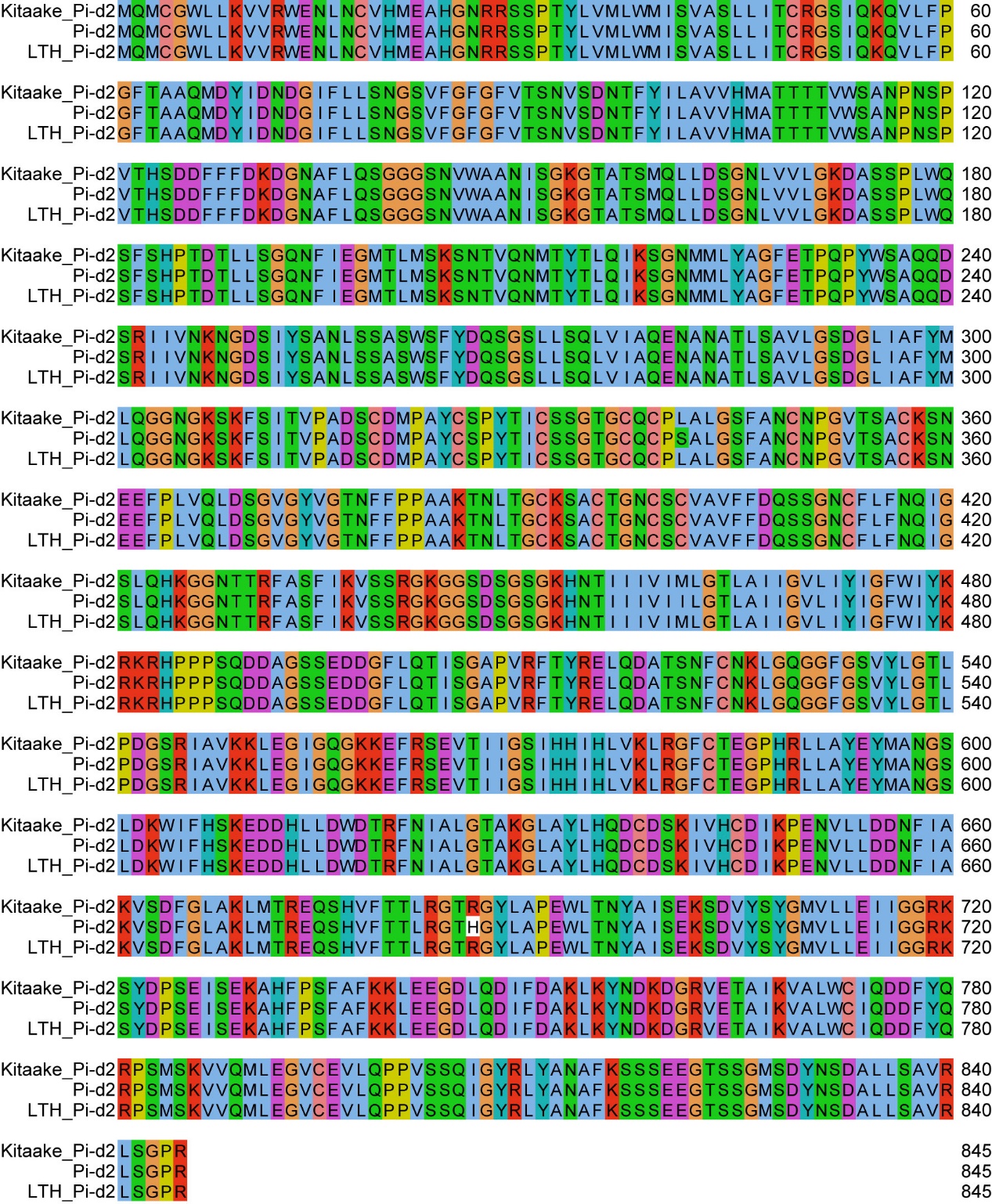


**Figure S15** Alignment of the candidate LTH gene to *OsBsr-k1*


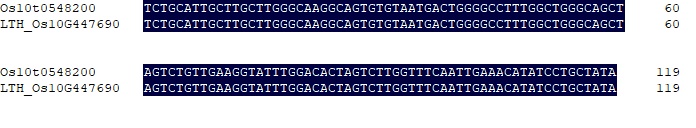


**Figure S16** Distribution of Nanopore read length


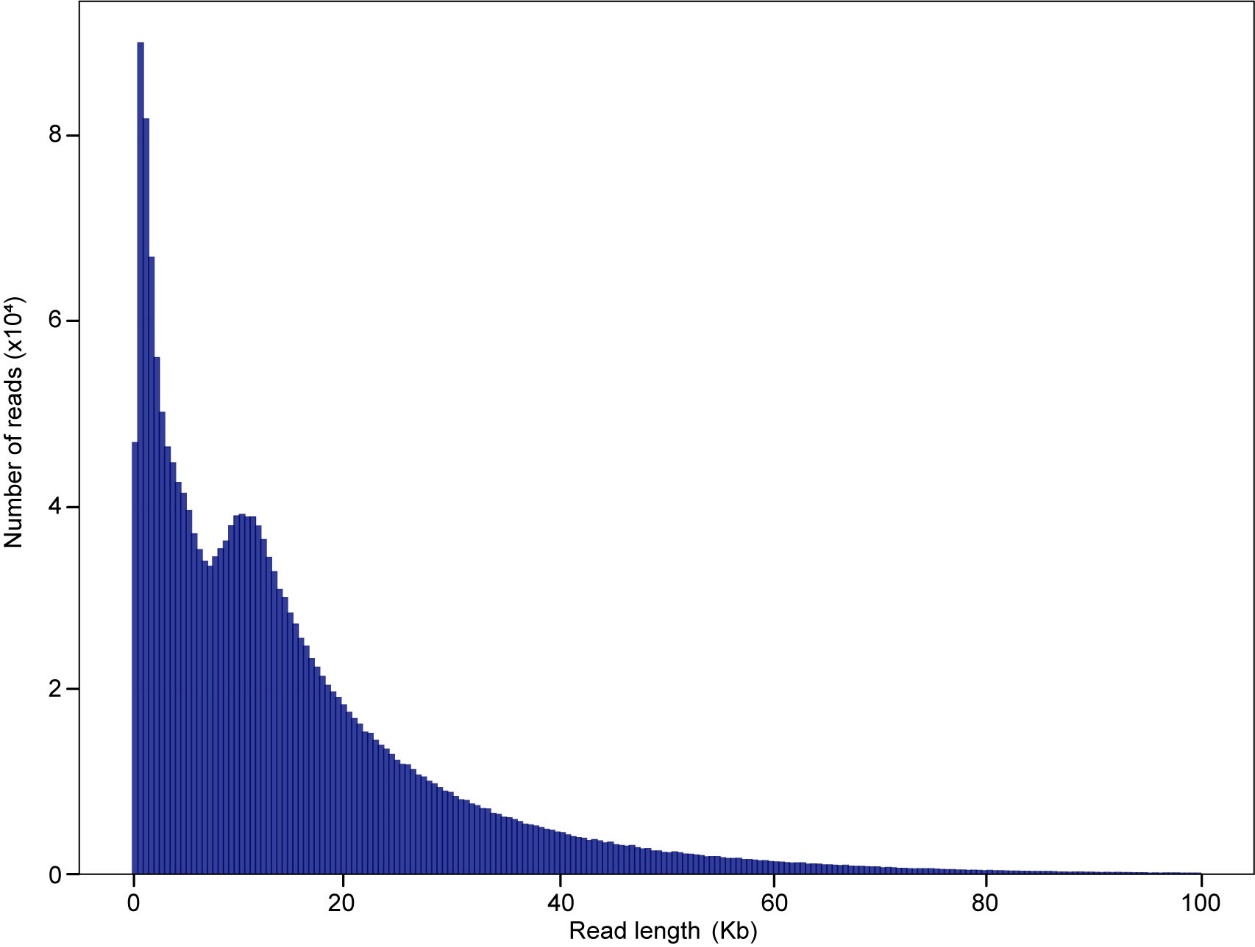

Supplement: Supplementary data 1 [file mmc1.docx]
